# Divergent selection along elevational gradients promotes genetic and phenotypic disparities among small mammal populations

**DOI:** 10.1002/ece3.5273

**Published:** 2019-05-28

**Authors:** Anderson Feijó, Zhixin Wen, Jilong Cheng, Deyan Ge, Lin Xia, Qisen Yang

**Affiliations:** ^1^ Key Laboratory of Zoological Systematics and Evolution, Institute of Zoology Chinese Academy of Sciences Beijing China

**Keywords:** altitude‐induced adaptation, isolation‐by‐environment, landscape ecology, mountain biota, phenotypic divergence, predation

## Abstract

Species distributed along mountain slopes, facing contrasting habitats in short geographic scale, are of particular interest to test how ecologically based divergent selection promotes phenotypic and genetic disparities as well as to assess isolation‐by‐environment mechanisms. Here, we conduct the first broad comparative study of phenotypic variation along elevational gradients, integrating a large array of ecological predictors and disentangling population genetic driver processes. The skull form of nine ecologically distinct species distributed over a large altitudinal range (100–4200 m) was compared to assess whether phenotypic divergence is a common phenomenon in small mammals and whether it shows parallel patterns. We also investigated the relative contribution of biotic (competition and predation) and abiotic parameters on phenotypic divergence via mixed models. Finally, we assessed the population genetic structure of a rodent species (*Niviventer confucianus*) via analysis of molecular variance and F_ST_ along three mountain slopes and tested the isolation‐by‐environment hypothesis using Mantel test and redundancy analysis. We found a consistent phenotypic divergence and marked genetic structure along elevational gradients; however, the species showed mixed patterns of size and skull shape trends across mountain zones. Individuals living at lower altitudes differed greatly in both phenotype and genotype from those living at high elevations, while middle‐elevation individuals showed more intermediate forms. The ecological parameters associated with phenotypic divergence along elevation gradients are partly related to species' ecological and evolutionary constraints. Fossorial and solitary animals are mainly affected by climatic factors, while terrestrial and more gregarious species are influenced by biotic and abiotic parameters. A novel finding of our study is that predator richness emerged as an important factor associated with the intraspecific diversification of the mammalian skull along elevational gradients, a previously overlooked parameter. Population genetic structure was mainly driven by environmental heterogeneity along mountain slopes, with no or a week spatial effect, fitting the isolation‐by‐environment scenario. Our study highlights the strong and multifaceted effects of heterogeneous steep habitats and ecologically based divergent selective forces in small mammal populations.

## 1 INTRODUCTION

In the era of rapid changes, predicting how species will respond to human‐mediated habitat alterations has become a 21st‐century scientific pursuit, uniting ecologists, evolutionary biologists, and conservationists (Fox, 2018). Anticipated climatic changes are expected to vary widely, leading to novel habitat–animal interactions and divergent selective forces (Bellard, Bertelsmeier, Leadley, Thuiller, & Courchamp, 2012; Keller, Alexander, Holderegger, & Edwards, 2013). Regarding this scenario, elevational gradients offer one of the best natural systems for understanding species performance under heterogeneous environmental constraints (Keller et al., 2013). Species distributed along mountain slopes, which face conspicuous climatic and steep vegetation changes on a short geographic scale, are of particular interest for testing how ecologically based divergent selection promotes phenotypic divergence and shape genetic structure across populations (Rosenblum, 2006).

Divergent natural selection, briefly defined as selection arising from uneven ecological conditions that promote phenotypic and genetic shifts among populations, is a key source of biological diversity (Nosil, Harmon, & Seehausen, 2008). As altitude increases, steep environmental changes promote spatially dynamic biotic and abiotic interactions. Such mixed effects of contrasting climatic variables on a short scale reflect marked heterogeneous vegetation zonation along mountain slopes, in some cases mirroring tropical–temperate forest transitions. For example, in southeastern Asian mountains, evergreen broadleaf forests occur at 1,000 m, and alpine landscapes occur at 3,500 m (Ohsawa, 1991, 1993). Individuals living in distinct elevation zones are thus expected to show phenotypic and genetic disparities owing to strong divergent ecological selection (Branch, Jahner, Kozlovsky, Parchman, & Pravosudov, 2017; Kawecki & Ebert, 2004; Keller et al., 2013; Ohsawa & Ide, 2008). In contrast, elevational gradients over a small geographic scale may facilitate migration, especially in species with a high dispersal capacity, thereby constraining morphological and genetic divergence (Branch et al., 2017; Kawecki & Ebert, 2004; Keller et al., 2013; Nosil et al., 2008; Ohsawa & Ide, 2008; Sexton, Hangartner, & Hoffmann, 2014; Waterhouse, Erb, Beever, & Russello, 2018). Thus, a tradeoff between the strength of divergent selective forces and dispersal patterns leads to distinct scenarios: a generalist phenotype and a panmictic population distributed across elevation zones or divergent phenotypes and structured genetic populations in each elevation zone (Allendorf & Luikart, 2007; Kawecki & Ebert, 2004; Nosil et al., 2008; Ohsawa & Ide, 2008).

Previous studies have pointed out that phenotypic and genetic divergence between animal populations along elevational gradients is not a rare phenomenon (reviewed by Keller et al., 2013). Intraspecific morphological shifts have been detected in numerous species of arthropods, lizards, frogs, and even small birds and mammals (Branch et al., 2017; Caro, Caycedo‐Rosales, Bowie, Slabbekoorn, & Cadena, 2013; Grieco & Rizk, 2010; Keller et al., 2013). However, whether this pattern is pervasive across groups and traits remains unclear because the majority of related studies have focused on low‐vagility animals (Keller et al., 2013) and phenotypic features with clear adaptive value along altitudinal gradients (e.g., cold and desiccation tolerance).

While morphological shifts have been widely assessed, their associated factors remain mostly speculative. Studies on the effects of climatic variation have mainly focused on temperature‐size correlations, testing the validity of Bergman's and Allen's rules along altitudinal gradients (Brehm & Fiedler, 2004; Du et al., 2017; Freeman, 2017; Gutiérrez‐Pinto et al., 2014). How competition and predation shape species morphology across elevation zones remains an open question. Increasing altitude leads to a reduced area, enhanced spatial isolation, and relaxed predation risk, similar to the patterns observed in insular systems (Körner, 2007). Considering well‐established island hypotheses, we would expect biotic interactions to play an important role in shaping morphological diversity in mountains as they do in islands (Heaney, 1978; Itescu et al., 2018; Michaux, Bellocq, Sarà, & Morand, 2002). To date, no study has investigated the combined effect of biotic and abiotic traits on phenotypic divergence on mammals along elevational gradients (Keller et al., 2013; Merilä & Hendry, 2014).

Further insights into divergent ecological selection can be derived from landscape genetic studies (Allendorf & Luikart, 2007; Kawecki & Ebert, 2004; Merilä & Hendry, 2014; Ohsawa & Ide, 2008). The presence of animals (particularly those with high vagility) in contrasting habitats on a short geographic scale makes such animals attractive candidates with which to test for ecologically induced genetic alterations. Isolation by environment (IBE) applies when the genetic variation among populations is mostly explained by climatic factors rather than spatial factors (Wang & Bradburd, 2014; Wang & Summers, 2010). Thus, the existence of limited gene flow between mammalian populations across mountain zones would indicate a strong role of heterogeneous landscapes in shaping the genetic structure of natural populations (Branch et al., 2017; Kierepka & Latch, 2015; Wang & Bradburd, 2014).

In this study, we aimed to obtain a comprehensive understanding of the effects of divergent selective forces along elevational gradients on genetic and phenotypic disparities in small mammals through three complementary steps. First, we analyzed nine species (shrews, moles, and rodents) distributed over a wide altitudinal range and with distinct ecological attributes to assess whether phenotypic divergence is a common phenomenon in small mammals and whether it shows consistent parallel patterns. We chose skull morphology (size and shape) as a surrogate of overall morphological diversity as it is associated with the main sensory systems related to environmental perception and reflects changes in the dietary and ecological interactions across populations (Klaczko, Sherratt, & Setz, 2016; Monteiro, Lessa, & Are, 1999; Nogueira, Peracchi, & Monteiro, 2009). Second, using a large array of biotic and abiotic parameters, we investigated the ecological factors associated with the intraspecific diversification of the mammalian skull along elevational gradients. Our goal here was to uncover the relative contribution of abiotic and biotic traits associated with phenotypic variation across ecologically distinct species. Finally, we assessed the population genetic structure of a widespread rodent species along three mountain slopes of southwest China and tested the IBE hypothesis, thereby disentangling the effects of climatic and spatially driven factors.

Given the dynamic habitat–animal interactions and strong divergent selection along elevational gradients, we hypothesized that species would show marked phenotypic disparity between populations inhabiting distinct altitude zones, regardless of their phylogenetic and ecological features. In contrast, considering species' inherent life‐history attributes, we predicted inconsistent main drivers of morphologic divergence across taxa (e.g., that desert dwellers would be more affected by variation in precipitation than would forest dwellers). Because of the large home range and generalist habits of our focal species, we expected weak or absent genetic structure along mountain slopes.

## 2 MATERIALS AND METHODS

### 2.1 Focal species and phenotypic traits

Nine mammal species (six rodents, two shrews, and one mole) distributed over a large altitudinal range and representing distinct ecological niches were selected to assess phenotypic variation (size and shape of the skull) across elevational gradients (Table 1). All specimens are housed in the mammal collection of the Institute of Zoology of the Chinese Academy of Science, Beijing, China. Specimens were sampled from the same mountain (*Soriculus*) or along altitudinal gradients with similar climatic conditions. In the last case, to minimize putative effects of distinct evolutionary history, we selected individuals from closely genetic lineages (Cheng et al., 2017; Ge et al., 2018; He, Hu, Chen, Li, & Jiang, 2016; Liu et al., 2018; Lv et al., 2018).

**Table 1:** Species of shrews, rodents, and moles used in this study, showing the sample size per elevation zone, species total elevation range, elevation range of the samples, and natural history attributes

Order	Family	Species	Elevation zone			Species elevation range (m)	Elevation range sampled (m)	Primary habitat	Primary diet	Life mode
			Low	Mid.	High					
Eulipotyphla	Soricidae	*Anourosorex squamipes*	55	18	–	1,200–3,000	1,123–2,837	Montane forests	Insectivorous	Fossorial
		*Soriculus nigrescens*	8	17	7	1,000–4,300	2,760–4,194	Conifer forests/Alpine	Insectivorous	Semifossorial
	Talpidae	*Uropsilus soricipes*	13	8	5	Up to 2,900	1,340–2,878	Conifer and deciduous forests	Insectivorous	Terrestrial
Rodentia	Cricetidae	*Eothenomys melanogaster*	16	10	–	Up to 3,000	103–1,944	Montane forests	Herbivorous	Terrestrial
	Dipodidae	*Allactaga sibirica*	32	38	21	Up to 3,500	166–2,970	Dry open areas/Desert/Semideserts	Omnivorous	Terrestrial
		*Dipus sagitta*	7	44	29	Up to 3,200	241–3,183	Deserts/Semideserts	Omnivorous	Terrestrial
	Muridae	*Apodemus chevrieri*	19	11	5	600–4,000	1,210–3,556	Montane forests	Omnivorous	Terrestrial
		*Apodemus ilex*	36	26	13	2,000–4,000	2,472–3,891	Montane forests	Omnivorous	Terrestrial
		*Niviventer confucianus*	37	11	8	150–4,000	1,210–3,430	Forests/Open lands	Omnivorous	Terrestrial

The phenotype was abstracted from the shape and size of the skull of 494 adult individuals using geometric morphometric techniques. A list of the specimens studied is provided in Appendix S1. The ventral view of the skull of adult specimens of a similar age (degree of tooth wear) was photographed with a Canon EOS Rebel t3i digital camera equipped with a 100‐mm fixed lens following standardized protocols. Landmarks on the left side of the skull were recorded from the digitized pictures using tpsDig version 2.3 (Rohlf, 2017). Landmark configuration was defined to reflect changes in the main sensory anatomical structures, such as the tympanic bulla, teeth, palate, and orbits (Table S2). The coordinates of each landmark were aligned, and the effects of location, orientation, and scale were removed through a generalized Procrustes analysis (GPA) using *geomorph* package version 3.0.5 (Adams, Collyer, Kaliontzopoulou, & Sherratt, 2017) in R software (R Development Core Team, 2017). Generalized Procrustes analysis generates centroid size values and aligned Procrustes coordinates (shape information) for each individual. The former can be defined as the square root of the summed squared distances between all landmarks and their centroid (Mitteroecker, Gunz, Windhager, & Schaefer, 2013) and is used here as an indicator of individual size. No sexual dimorphism in skull shape or size was found in the nine species examined (Table S3); therefore, we pooled males and females in the following analyses.

### 2.2 Ecological parameters

**Figure 1:**
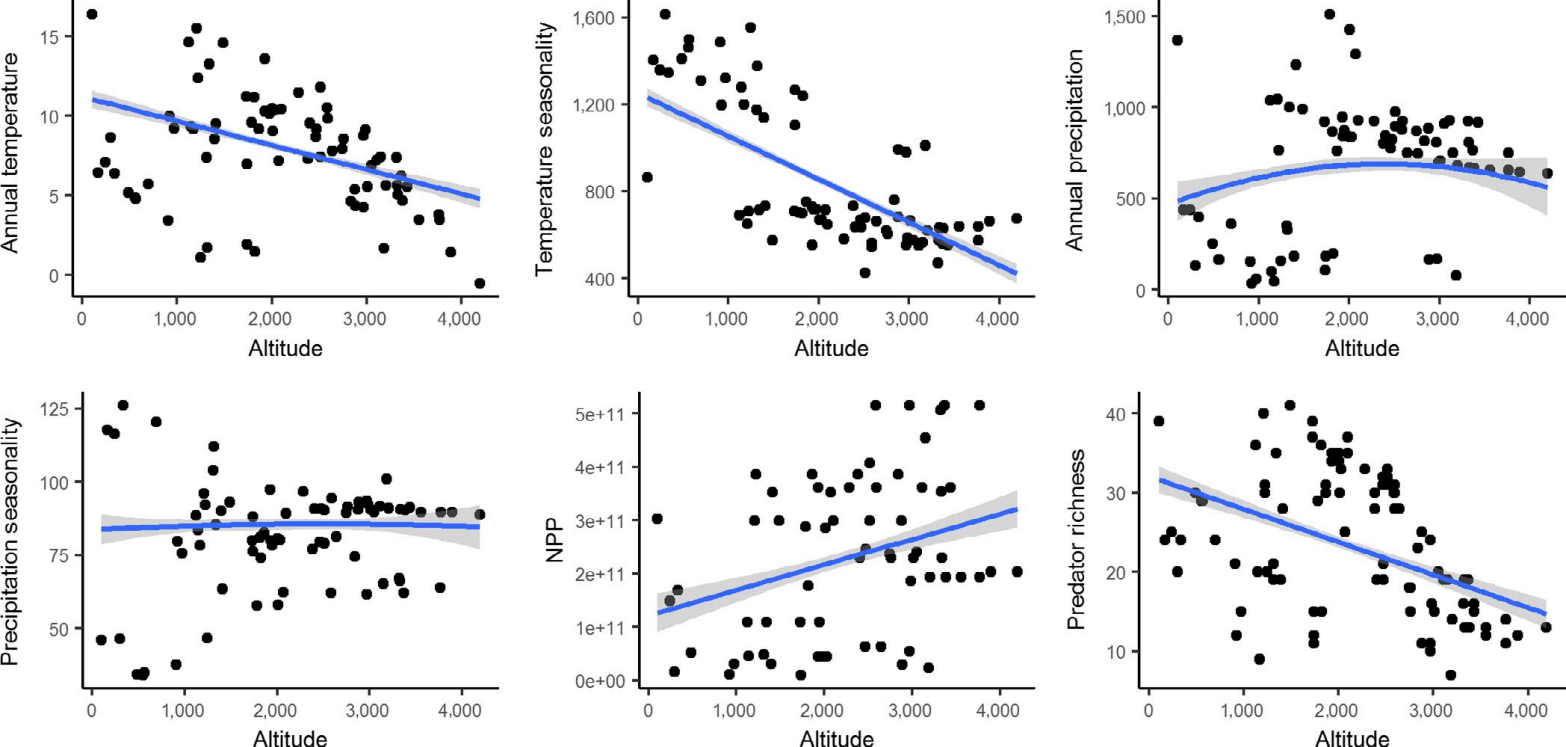
Ecological parameter trends along elevational gradients sampled in this study. Dots represent individual collection localities

We quantified the effects of climate and biotic interactions on skull phenotypic changes of mammals using a large array of biotic and abiotic predictors. The topographical dataset was obtained from the WorlClim website and generated from NASA's Shuttle Radar Topography Mission (https://www2.jpl.nasa.gov/srtm/). Based on the geographic coordinates of each individual, we extracted the altitude information via the *raster* R package (Hijmans, 2017). Five climate traits were used: annual mean temperature, temperature seasonality, annual precipitation, precipitation seasonality, and net primary productivity (NPP). Net primary productivity was obtained from the NASA Socioeconomic Data and Applications Center (Imhoff & Bounoua, 2006; Imhoff et al., 2004). Precipitation and temperature traits were extracted from WordClim version 2 (http://worldclim.org/version2) at a 30‐s (~1 km^2^) spatial resolution (Fick & Hijmans, 2017) via the *raster* package. Figure 1 summarizes the trends in ecological parameters along elevational gradients encountered by our focal species.

To assess the effects of competition (intra‐ and interspecific), we used the database of sampling collections of the mammal group of the Institute of Zoology of the Chinese Academy of Science. This unique database includes specimens of small mammals systematically collected from several mountains in China. Detailed descriptions of the sampling protocols can be found in Wu et al. (2013) and Wen et al. (2018). For each specimen's collection locality, we computed the number of individuals of a given selected species and the species richness recorded in a 1‐km buffer. We used these two parameters as proxies of intraspecific and interspecific competition, respectively. Although this counting method (Dueser & Hallett, 1980; Krebs, 1966) likely underestimates the overall abundance and local richness of small mammals, the results from standardized sampling protocols applied by our group can still be used in a comparative way (Krebs et al., 2011; Pacheco et al., 2013).

To evaluate the influence of predation risk on phenotypic variation, we compiled a list of predators of small mammals (i.e., carnivorous mammals, snakes, and predatory birds) in China (Hoyo, Elliott, & Sargatal, 1999; Smith & Xie, 2013). The final list comprised 136 species, specifically, 62 birds, 45 mammals, and 29 snakes (Table S4). Then, we compiled the geographic distribution of each predator based on the IUCN species range (IUCN, 2017), taking into account the altitudinal range limits of each species. For each collection locality of our focal species, we computed the number of possible predators (predator richness) in a 1‐km buffer. In a complementary approach, we added a parameter including only predator species with small terrestrial mammals as a primary dietary component. This second list included 45 birds, 29 mammals, and 23 snakes. In general, both approaches generated similar results. To ensure that our predator list are not overestimated, we compared our database with published checklists available for some of the mountains studied. For example, we estimated a total of 13 mammalian predators that have terrestrial mammals as their primary diet in Wolong Mountain. In the same area, Shi et al. (2017) based on a large camera‐trap survey recorded 13 species of Carnivora that prey small mammals. Therefore, we are confident with our predator database.

### 2.3 Molecular sampling

**Figure 2:**
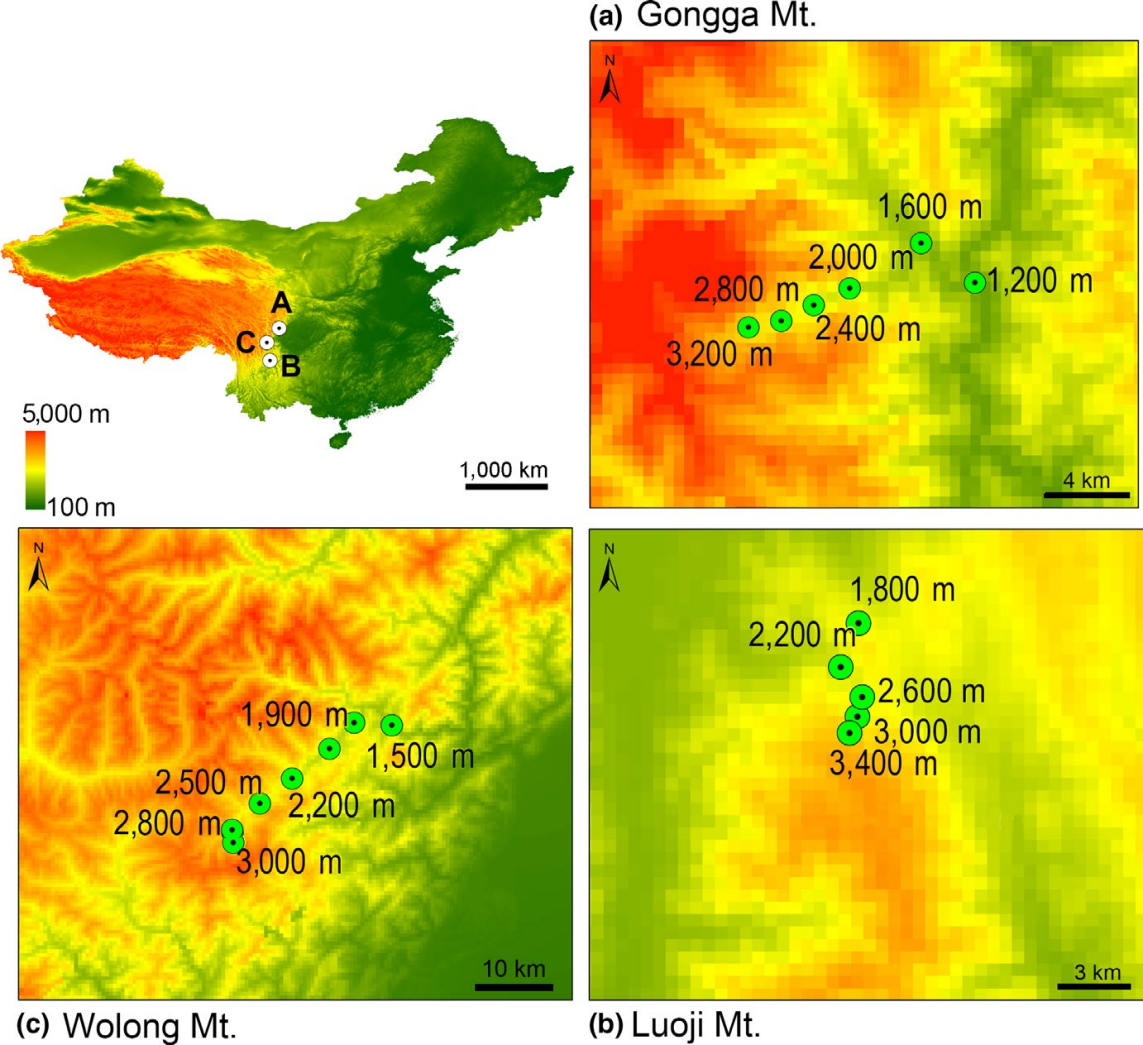
Genetic sampled populations of *Niviventer confuncianus* in Gongga (a), Luoji (b), and Wolong (c) Mountains, Sichuan, China, showing the altitude of each collecting locality

To explore the genetic structure of small mammal populations distributed along elevational gradients, we selected the Confucian white‐bellied rat (*Niviventer confucianus*) as a model due to its wide elevational range (150–4,000 m), broad climatic tolerance, and relatively high dispersal capability (average home range of approximately 2,200 m^2^; Ge et al., 2018; Shen, Bao, Xu, Wei, & Liu, 2011). DNA samples were obtained from 252 individuals collected along linear transects on three mountain slopes (Gongga, Luoji, and Wolong Mountains) in Sichuan Province, South China (Figure 2). The protocols of DNA extraction and sequencing are detailed in Ge et al. (2018). We used the mitochondrial cytochrome b (cytb) gene (1,132 bp) because it presents a high level of haplotype diversity in this species (Ge et al., 2018). All sequences are available from GenBank (Table S5).

On Gongga Mountain, we obtained complete cytb sequences (1,132 bp) from 160 individuals comprising six populations covering an altitudinal range of 2,000 m (Figure 2). The median Euclidian distance between sampling localities was 3.2 km (range: 2.69–6.9 km). On Luoji Mountain, we obtained cytb sequences from 69 specimens representing five populations covering an altitudinal range of 1,600 m, and the median Euclidian distance between sampling localities was 1.6 km (range from 1.34–6.8 km). On Wolong Mountain, we obtained cytb sequences from 23 individuals comprising six populations distributed at an altitudinal range of 1,500 m with a median Euclidian distance between collecting localities of 11.2 km (range from 0.62–19 km; Table 2). For all mountains, we divided our sample into three elevation zones (low, middle, and high) as detailed in Table 2. Information on the climate and vegetation of Gongga, Luoji, and Wolong Mountains is given in Wu et al. (2013), Ma et al. (2010), and Wen et al. (2017), respectively.

**Table 2:** Sample size of cytb sequences of *Niviventer confucianus* populations (Pop.) used in this study according to the elevation zone and altitude in three mountains of China

Elevation zone	Gongga Mt.	Luoji Mt.	Wolong Mt.
Low	Pop. 1: 1,200 m (*n* = 22)	Pop. 1: 1,800 m (*n* = 3)	Pop. 1: 1,500 m (*n* = 5)
	Pop. 2: 1,600 m (*n* = 39)		Pop. 2: 1,900 m (*n* = 4)
	Pop. 3: 2,000 m (*n* = 57)		
Middle	Pop. 4: 2,400 m (*n* = 15)	Pop. 2: 2,200 m (*n* = 4)	Pop. 3: 2,200 m (*n* = 3)
	Pop. 5: 2,800 m (*n* = 20)	Pop. 3: 2,600 m (*n* = 23)	Pop. 4: 2,500 m (*n* = 2)
High	Pop. 6: 3,200 m (*n* = 7)	Pop. 4: 3,000 m (*n* = 29)	Pop. 5: 2,800 m (*n* = 6)
		Pop. 5: 3,400 m (*n* = 10)	Pop. 6: 3,000 m (*n* = 3)
Total	160	69	23

### 2.4 Statistical analyses

#### 2.4.1 Phenotypic comparison across elevation zones

To evaluate the phenotypic divergence across elevational gradients, we divided our samples into three bioclimatic zones (low, middle, and high elevation). The delimitation of zones combined climatic and vegetation attributes and was based on Tang and Ohsawa (1997), Zhong, Zhang, and Luo (1999), and Li and Zhang (2010). The elevational range of each zone might vary among mountains as a reflection of distinct topographical and regional climatic conditions. This relationship explains the occurrence of some overlap between elevation zones across mountains. The lowest zone is characterized by evergreen broadleaf forests and occurs at elevations up to 2,900 m. The middle zone is characterized by a predominance of deciduous tree species and ranges from 1,300 to 3,400 m. The high zone includes coniferous trees and shrub‐grassland alpine landscapes spanning 2,800–4,200 m in our dataset.

To test for phenotypic divergence among elevation zones, we conducted analyses of variance using Procrustes coordinates for shape and centroid size for size in the *geomorph* R package with 10,000 permutations. In addition, we carried out pairwise comparisons between mean shapes and performed Tukey's honestly significant difference (HSD) test to assess the significance of morphological differences between pairs of elevation zones. We also explored size and shape trends across species to determine whether there was a consistent overall pattern. The relationship between size (centroid size) and altitude was visualized with scatterplots highlighting trends in each elevation zone. Skull shape deformation between zones was assessed via between‐group principal component analysis (BgPCA; Mitteroecker & Bookstein, 2011) using *Morph* R package (Schlager, 2017).

#### 2.4.2 Phenotypic–ecological interaction analyses

We assessed the combined effects of our ecological parameters (and quadratic terms when the relationship was nonlinear) on skull phenotypes by creating a set of competing models ranked by the second‐order corrected Akaike information criterion (AICc) via the *MuMln* R package (Barton, 2018) and tested them by using mixed hierarchical models in the *lme4* R package (Bates, Maechler, Bolker, & Walker, 2015). In all models, site was included as a random effect to account for uneven sample sizes among collection localities (Harrison et al., 2018; Paterson, 2003; Schielzeth & Forstmeier, 2008). We prevented overparameterization by limiting the number of predictors in a single model based on the sample size (Burnham & Anderson, 2002; Grueber, Nakagawa, Laws, & Jamieson, 2011; Harrell, 2001; Harrison et al., 2018). The importance of each parameter in models with similar likelihoods was summarized using the full model‐average approach (Burnham & Anderson, 2002; Grueber et al., 2011) considering only models with a ∆AICc ≤ 6 following Richards (2008).

Summarizing shape information in univariate terms might lead to a loss of information (Fontaneto et al., 2017). Therefore, we use two variables to provide a more complete description of shape in the mixed analyses. The first univariate term consisted of the score of regression of Procrustes coordinates on the log of centroid size, which has been shown to be an effective descriptor of intraspecific shape variation (Drake & Klingenberg, 2008; Maestri et al., 2016; Martinez et al., 2018). The second variable was the first principal component of the Procrustes coordinates, which summarized most of the shape variation. In this case, to account for the allometry effect, we added the scaled centroid size as an independent covariate in our models.

Spatial autocorrelation is an inherent issue in interpopulation studies that can potentially bias statistical parameters and inflate the type I error (Chun & Griffith, 2011; Diniz‐Filho, Bini, & Hawkins, 2003; Perez, Diniz‐Filho, Bernal, & Gonzalez, 2010). To account for spatial dependence in our statistical models, we applied the eigenvector spatial filtering method (detailed in Griffith, 2003), which introduces a set of positive eigenvectors (spatial filters) extracted from the spatial weight matrix as fixed factors into regression models. The spatial filters were obtained via the *spdep* R package (Bivand & Piras, 2015) following the protocol described by Griffith and Chun (2014). Finally, we ran Moran's I test to ensure that the final residuals of our mixed models lacked significant spatial autocorrelation.

#### 2.4.3 Population genetic analyses

DNA sequences were aligned using the ClustalW algorithm implemented in MEGA 7.0 (Kumar, Stecher, & Tamura, 2016). To assess the patterns of genetic diversity among elevation zones, we calculated the nucleotide diversity, the mean number of pairwise differences, the number of haplotypes, and the haplotype diversity for each population using DnaSP 6.11.01 (Rozas et al., 2017). We tested the genetic difference among populations based on haplotype frequencies via a chi‐squared test with 1,000 permutations implemented in DnaSP. Population genetic structure along elevation zones was measured via analysis of molecular variance (AMOVA) based on haplotype diversity (Excoffier, Smouse, & Quattro, 1992) using Arlequin 3.5.2 software (Excoffier & Lischer, 2010), and the significance was assessed via 10,000 permutations. In a fine‐scale approach, we calculated the *F*_ST_ between pairs of populations (Figure 2) to explore patterns of genetic differentiation along mountain slopes, and significance levels were obtained through 10,000 permutations in Arlequin.

To investigate the correlation between genotype and spatial environmental components and assess whether the putative genetic structure was caused by random (isolation by distance) or nonrandom (IBE) mechanisms, we performed two complementary tests (Kierepka & Latch, 2015). First, we carried out a Mantel test with 1,000 permutations to explore the correlation between genetic and geographic distances for each mountain using the vegan R package (Oksanen et al., 2018). To disentangle the effects of spatial and climatic variables on the landscape genetic structure along mountain slopes, we carried out a redundancy analysis (RDA) in the vegan package. Redundancy analysis is a robust test with low type I error used to evaluate environmental effects on population genetic structure (Forester, Lasky, Wagner, & Urban, 2018; Kierepka & Latch, 2015). We used the scores of the principal coordinate analysis (PCoA) of the Euclidean distance matrix as dependent variables to summarize the genetic divergence among individuals. All PCoA axes were retained for this analysis. The explanatory variables included the spatial coordinates (latitude and longitude) and the six climatic parameters previously described. We assessed the isolated and combined contributions of each set of variables (spatial and climatic) by running five models: (a) a full model with climatic and spatial coordinates as explanatory variables, (b) a model with only climatic variables, (c) a model with only spatial variables, (d) a model with the isolated effect of climatic variables and controlling for spatial influence, and (e) a model with the isolated effect of spatial structure and controlling for climatic parameters.

## 3 RESULTS

### 3.1 Phenotypic comparison across elevation zones

Significant cranial differences among elevation zones were detected in all species examined (Table 3). Decomposition of skull form components revealed significant shape differences among the zones in eight species, while size diverged in five species. However, elevational zonation tended to explain a larger proportion of variance in size (9%–49%) than in shape (3%–19%; Table 3). Further inspection using pairwise and Tukey's HSD tests revealed that animals inhabiting low altitudes were differentiated from those living at middle and high altitudes to a greater extent than individuals from middle altitudes were differentiated from those at high elevations (Table S6).

**Table 3:** Phenotypic disparity among populations living at distinct elevation zones, showing the results of Procrustes ANOVA for shape (Procrustes coordinates) and size (centroid size)

Species	Form	*df*	*R* ^2^	*F*	*p*
*Anourosorex squamipes*	Shape	1,72	0.03	2.34	**0.004**
	Size	1,72	0.20	18.43	**0.0001**
*Soriculus nigrescens*	Shape	2,31	0.11	1.82	**0.009**
	Size	2,31	0.01	0.24	0.77
*Uropsilus soricipes*	Shape	2,25	0.13	1.89	**0.007**
	Size	2,25	0.04	0.53	0.59
*Eothenomys melanogaster*	Shape	1,25	0.08	2.04	**0.01**
	Size	1,25	0.49	23.46	**0.0001**
*Allactaga sibirica*	Shape	2,90	0.18	10.6	**0.0001**
	Size	2,90	0.25	15	**0.0001**
*Dipus sagitta*	Shape	2,79	0.19	9.43	**0.0001**
	Size	2,79	0.02	0.84	0.44
*Apodemus chevrieri*	Shape	2,33	0.11	2.41	**0.012**
	Size	2,33	0.16	3.04	**0.05**
*Apodemus ilex*	Shape	2,74	0.03	1.29	0.13
	Size	2,74	0.09	3.74	**0.02**
*Niviventer confucianus*	Shape	2,55	0.06	1.86	**0.007**
	Size	2,55	0.07	2.24	0.11

Note

Numbers in boldface represent significant values (*p* < 0.05 based on a permutation test with 10,000 randomizations).

**Figure 3:**
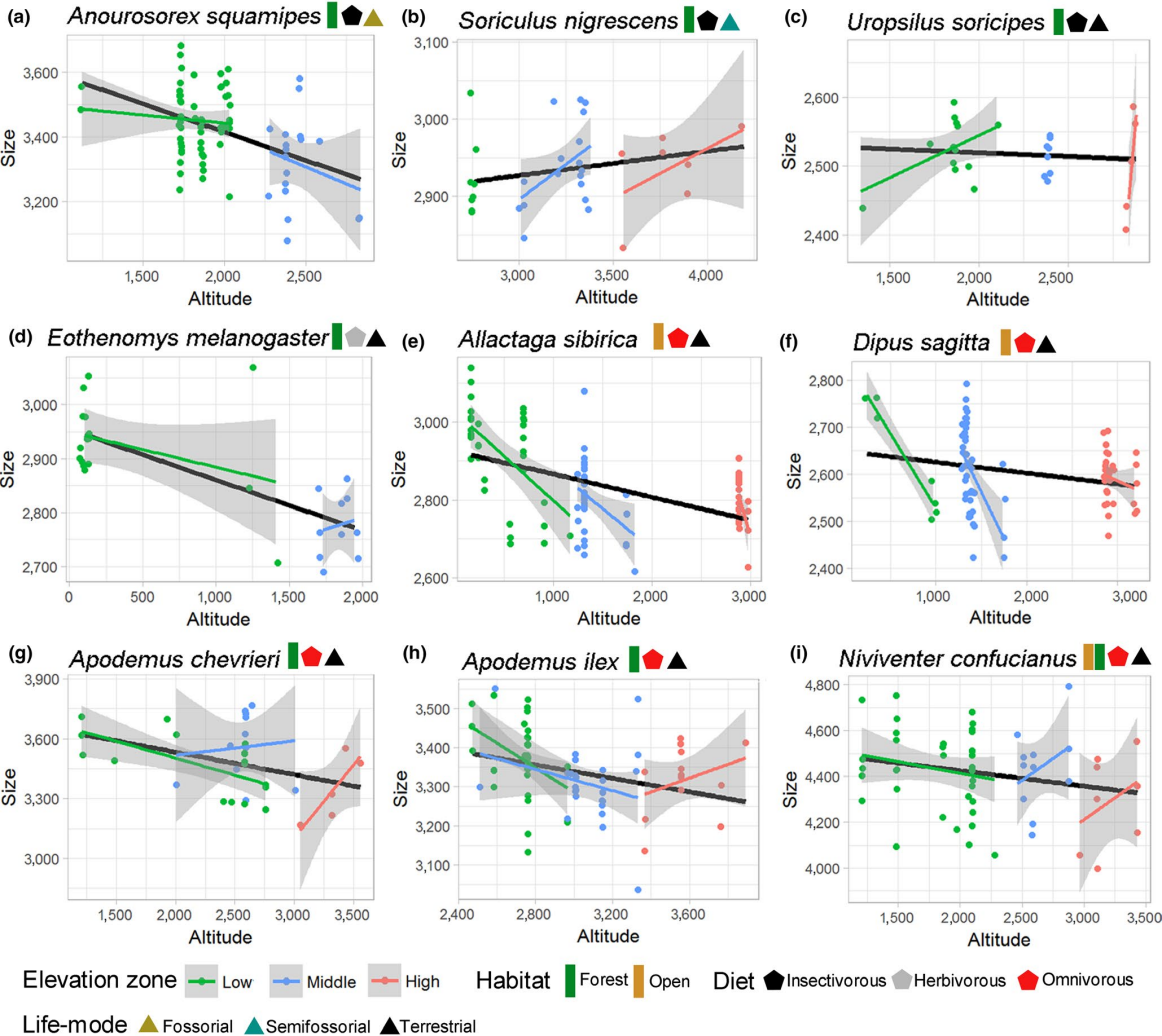
Patterns of size (centroid size) as function of altitude (meters) per elevation zone of shrews (a, b), moles (c), and rodents (d–i). Information on habitat, diet, and life mode of each species is shown in colored polygons. Dots represent individual centroid size. Lines represent the best fit linear regression of overall trend (black) and trend per elevation zone (colored) with standard deviation

**Figure 4:**
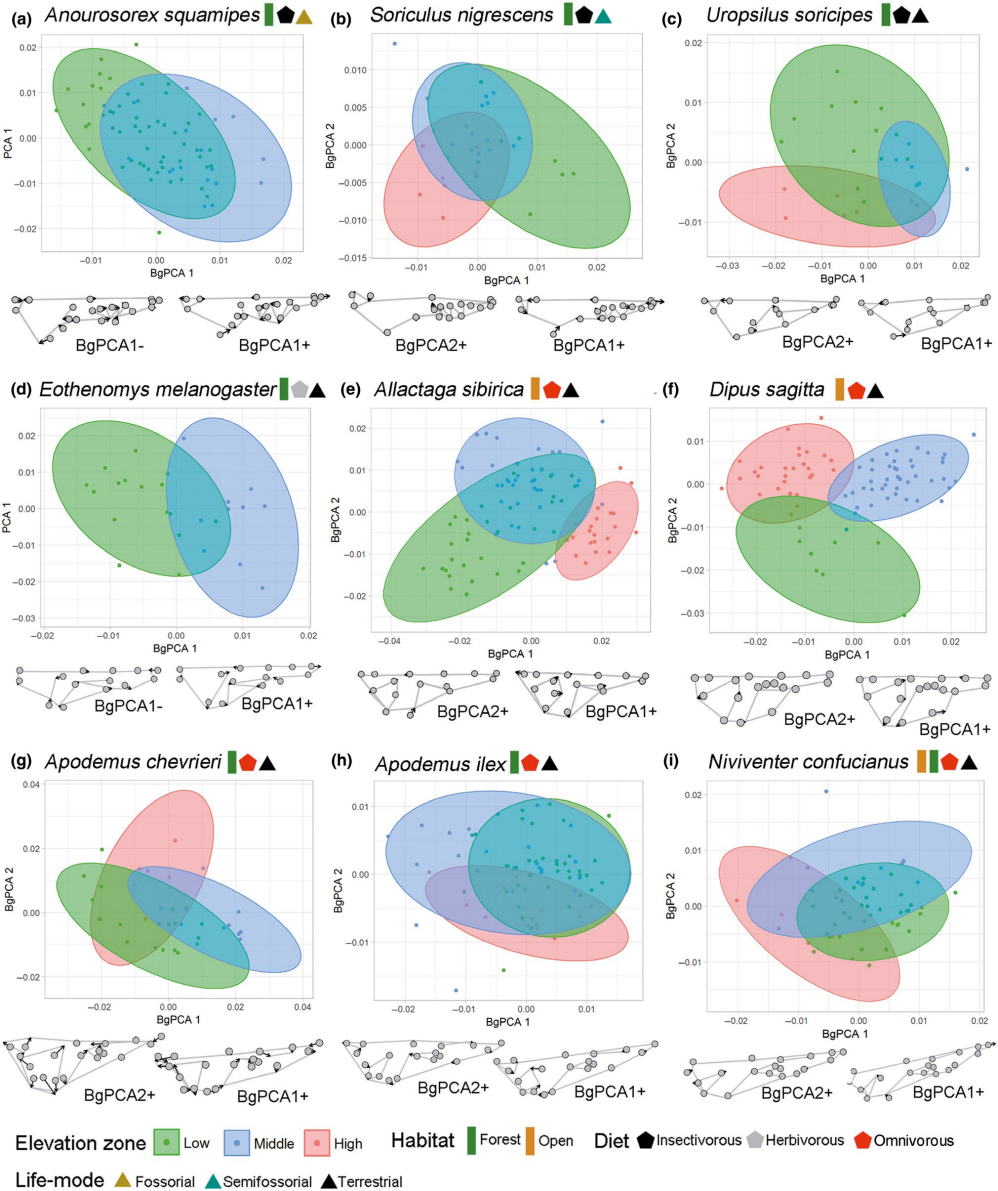
Between‐group PCA of skull shape (Procrustes coordinates) per elevation zone with 95% confidence ellipses of shrews (a, b), moles (c), and rodents (d–i). Information on habitat, diet, and life mode of each species is shown in colored polygons. Deformation grids show skull shapes at the extremes of each axis

Eight of nine species exhibited a weak‐to‐moderate decrease in size as a function of altitude (Figure 3), although this relationship was significant in only two species (*Anourosorex squamipes* [slope = −63.21, *p* = 0.04] and *Apodemus ilex* [slope = −46.05, *p* = 0.02]). We found a mixed pattern of size trends across elevation zones. Rodents exhibited a marked decrease in size as altitude increased at low elevations, while shrews and moles were not affected or exhibited a weak positive correlation. Both groups presented contrasting patterns at middle elevations, and five of the seven species presented positive trends in size at high elevations (Figure 3). Skull shapes overlapped somewhat among all three elevation zones, but the shapes of individuals from middle elevations overlapped to a greater degree with those of individuals from low altitudes (Figure 4). Mammals from high altitudes tended to exhibit a wider braincase and larger tympanic bulla than those from lower altitudes, although most anatomical structures (rostrum, orbit, palate, and teeth) showed variable shape patterns across species (Figure 4).

### 3.2 Ecological factors associated with phenotypic changes along elevational gradients

Hierarchical regressions of skull phenotype on ecological predictors uncovered an inconsistent combination of factors that best explained phenotypic variation across taxa (Table 4). The skull of shrews was mainly affected by environmental traits, whereas rodents and moles responded to both biotic and abiotic interactions. Size variation along elevational gradients was explained by equally important multiparameters in most cases, while skull shape variation was correlated with one variable or a few variables (Table 4). Altitude was among the top variables in the selected models in terms of explaining size variation in all species examined, and its effects varied from weak to strong across taxa (Table 4). Among the biotic variables, predator richness had the most widespread effect across species and, in most cases, had a positive relationship with skull size (Table 4).

**Table 4:** Estimates (and percentage of relative importance) for each parameter from the full averaged models of biotic and climatic parameters on skull phenotype (size and shape) of small mammals along elevational gradients

Parameter	*Anourosorex squamipes*	*Soriculus nigrescens*	*Uropsilus soricipes*	*Eothenomys melanogaster*	*Allactaga sibirica*	*Dipus sagitta*	*Apodemus chevrieri*	*Apodemus ilex*	*Niviventer confucianus*
Size									
Intercept	**3,426**	**2,952.46**	**2,516.61**		**2,838.43**	**2,589.37**	**3,474.83**	**3,339.69**	**4,412.25**
Altitude	**−3.64 (80)**	1.16 (5)	−111.64 (52)	−1.79(4)	14.53 (21)	0.77 (5)*	4.84 (11)	−14.34 (21)	−3.59 (11)
Intra. Comp.	−0.01(<1)		0.22 (9)	**175.73 (1)**	**2.64 (11)**	**3.17 (6)**	−0.48 (3)	−0.1 (1)	1.63 (1)
Inter. Comp.	−0.05 (<1)		**6.36 (3)**		0.68 (4)		0.14 (1)	0.2 (3)	2.38 (8)
Pred. richn.	**33.63 (39)**		**19.88 (31)**		−0.51 (3)		**114 (94)**	35.39 (52)	5.05 (12)
Main pred. richn.	**27.86 (38)**		20.68 (33)				1.3 (7)	−2.9 (38)	3.25 (10)
NPP	**−6.67 (13)**	−3.16 (7)	0.33 (5)		−2.03 (7)	**3.02 (6)**	−2.46 (6)	−15.18 (37)	−2.34 (8)
Annual Temp.	**6.63 (12)**	−0.39 (2)	−147.2 (57)		0.2 (1)		−10.53 (18)	−9.41 (17)	−1.38 (11)
Temp. Seas.			−31.59 (64)		**70.42 (67)**		0.94 (3)	16.31 (36)	3.1 (9)
Annual Prec.	**0.34 (1)**	−0.57(2)	−**14.48 (45)**		2.07 (6)	**70.67 (94)**	−10.61 (19)	8.58 (25)	0.52 (4)
Prec. Seas.	**−0.21 (**1)	−0.54(2)	**12.89 (36)**		85.26 (69)*		5.26 (1)	26.94 (51)	**62.57 (85)**
Shape									
Intercept					0.001				
Altitude				**0.00004 (1)** a	**−0.009 (2)** b	0.0002(1)^b^ ^,^*			
Intra. Comp.									
Inter. Comp.			**0.004 (1)** a					**0.0002 (5)** b	
Pred. richn.							**0.001 (18)**		
Main pred. richn.	**0.0003 (1)** a						**0.0006 (8)**		
NPP	**−0.0004 (16)** a							**0.001 (24)** b	
Annual Temp.				**0.00005 (1)** a					
Temp. Seas.								**−0.0004 (9)** b	
Annual Prec.					**0.0008 (2)** b				
Prec. Seas.					**0.004 (3)** b ^,^*				

Note

Bold face indicates parameters considered significant, when confidence intervals (95%) did not include zero. Full statistics are presented in Table S7. All traits were standardized prior to analyses. Spatial filters were included as fixed factors to account for spatial autocorrelation. Inter. Comp.: interspecific competition; Intra. Comp.: intraspecific competition; Main pred. richn.: main predator richness (species that have small terrestrial mammals as their primary diet); NPP: net primary productivity; Prec., precipitation; Pred. richn.: predator richness; Seas.: seasonality; Temp.: temperature. Asterisks indicate that the quadratic term of the parameter was used.

a Represents the score of regression of Procrustes coordinates on the log of centroid size.

b Represents the first principal component of Procrustes coordinates.

### 3.3 Population genetic structure along mountain slopes

On all three mountains, the frequency of haplotypes (Gongga Mt.: *X* ^2^ = 288.01, *p* = 0.0001; Luoji Mt.: *X* ^2^ = 184.81, *p* = 0.000; Wolong Mt.: *X* ^2^ = 121.7, *p* = 0.000) differed significantly among populations. Similarly, AMOVA revealed significant population genetic structure (Gongga Mt.: Ф_ST_ = 0.038, *p* = 0.026; Luoji Mt.: Ф_ST_ = 0.14, *p* = 0.018; Wolong Mt.: Ф_ST_ = 0.40, *p* = 0.000). A large part of the genetic variation was attributed to variance within populations (59.9%–96.1%), whereas differences among elevation zones were low to moderate, albeit not significant (Gongga Mt.: 1.56%, *p* = 0.21; Luoji Mt.: 10.1%, *p* = 0.12; Wolong Mt.: 24.3%, *p* = 0.19; Table S8). Based on comparisons of pairs of populations sampled along mountain slopes, middle‐elevation populations had the highest nucleotide diversity (Table S9). In addition, the F_ST_ values indicated consistent and strong genetic differentiation (Gongga Mt.: *F*_ST_ = 0.26, *p* = 0.002; Luoji Mt.: *F*_ST_ = 0.43, *p* = 0.008; Wolong Mt.: *F*_ST_ = 0.59–0.77, *p* = 0.01) between animals inhabiting low (Gongga Mt.: 1,200 m; Luoji Mt.: 1,500 m; Wolong Mt.: 1,500 m) and high altitudes (Gongga Mt.: 3,200 m; Luoji Mt.: 3,000 m; Wolong Mt.: 2,800 m and 3,000 m). Comparisons between populations from low and middle (Gongga Mt.: *F*_ST_ = 0–0.02; Luoji Mt.: *F*_ST_ = 0.08–0.11; Wolong Mt.: *F*_ST_ = 0.12–0.87) and middle and high elevations (Gongga Mt.: *F*_ST_ = 0.10–0.15; Luoji Mt.: *F*_ST_ = 0.08–0.11; Wolong Mt.: *F*_ST_ = 0–0.14) revealed generally moderate‐to‐low genetic differentiation (Table S10). The genetic structure of *N. confucianus* was mainly driven by climatic variables (Table 5). The amount of genetic variance explained by our set of environmental parameters (up to 37%) was significant across all mountains, even when the spatial component was controlled for. In contrast, spatial parameters had either no effect (in Luoji Mountain: Mantel *r* = 0.02, *p* = 0.37) or a weak effect on the landscape genetic structure of *N. confucianus* (Table 5).

**Table 5:** Results of redundancy analysis (RDA) models to assess the contribution of climatic and spatial variables on *Niviventer confucianus* genetic structure along the mountain slopes in China

Models	*R* ^2^ adj	*p* value
Gongga Mountain		
Climatic + Spatial	0.05	**0.001**
*only* Climatic	0.05	**0.003**
*only* Spatial	0.02	**0.007**
Climatic (Spatial *controlled*)	0.02	**0.01**
Spatial (Climatic *controlled*)	0.01	0.07
Luoji Mountain		
Climatic + Spatial	0.16	**0.003**
*only* Climatic	0.16	**0.002**
*only* Spatial	0.008	0.24
Climatic (Spatial *controlled*)	0.15	**0.001**
Spatial (Climatic *controlled*)	0.08	**0.001**
Wolong Mountain		
Climatic + Spatial	0.37	**0.004**
*only* Climatic	0.37	**0.001**
*only* Spatial	0.19	**0.001**
Climatic (Spatial *controlled*)	0.17	**0.02**
Spatial (Climatic *controlled*)	0.03	0.18

Note

Numbers in boldface represent significant values (*p* < 0.05).

## 4 DISCUSSION

By comparing diverse phylogenetic and ecological groups of small mammals, we found consistent phenotypic disparity in cranial traits and marked genetic structure along elevational gradients. This finding, combined with previous evidence of life‐history and physiologically changes along mountain slopes (Keller et al., 2013), reinforces the strong and multifaceted effects of heterogeneous steep habitats on individuals' features.

The phenotypic pattern of individuals living at lower elevations differed greatly from that of individuals living at high elevations, which paralleled our genetic results. We can speculate that this morphogenetic divergence may be related to the pronounced ecological differences faced by populations at opposite ends of an elevational gradient (Caro et al., 2013; Kawecki & Ebert, 2004; Wang & Bradburd, 2014). This interpretation also explains the lower morphological and genetic differentiation of middle‐elevation individuals as they inhabit less contrasting environmental conditions and thus are exposed to weaker divergent selective forces (Allendorf & Luikart, 2007; Kawecki & Ebert, 2004). In addition, animals at middle altitudes are in closer contact with both low‐ and high‐altitude populations, which may favor intermediate forms able to exploit distinct elevation zones. In contrast, high‐altitude animals with higher levels of differentiation are less likely to colonizing other zones (Kawecki & Ebert, 2004). Although further studies focusing on the genes linked to the observed phenotypic changes are still needed (Merilä & Hendry, 2014), the parallel results obtained from the genetic and morphological approaches suggest that evolutionary mechanisms rather than plasticity alone are likely to contribute to the intraspecific diversification of the mammalian skull along elevational gradients (Boutin & Lane, 2014; Merilä & Hendry, 2014).

The higher genetic diversity of middle‐altitude populations of *N. confucianus* might reflect optimal ecological conditions faced by these individuals (Ohsawa & Ide, 2008). This hypothesis predicts that for species with elevational range centers at middle altitudes, as *N. confucianus* (Ge et al., 2018; Wen et al., 2017), peripheral populations at low and high altitudes experience suboptimal conditions, which reduce their niche breadth, their population size, and ultimately their genetic variation (Heino & Gronroos, 2014; Ohsawa & Ide, 2008; Wen et al., 2018). Therefore, we expect that species with distinct elevational range centers will show different patterns of genetic diversity along mountain slopes. Nevertheless, the absence of an effect or a weak effect of spatial traits on the population genetic structure of *N. confucianus* along the three mountain slopes points to more general patterns for small mammals, where nonrandom factors are the main driver of genetic variation (Garant, Forde, & Hendry, 2007; Keller et al., 2013; Kierepka & Latch, 2015). Accordingly, RDA revealed that environmental variables explained up to 17% of the genetic variance when geography was controlled for. These results fit an IBE scenario, especially considering that the distance between the genetically sampled populations was shorter than the individual dispersal capacity (Kierepka & Latch, 2015; Sexton et al., 2014; Wang & Bradburd, 2014).

Skull shape divergence appears to be a more common phenomenon than size divergence in mammals distributed across a wide elevational range, although, when present, the difference in size tends to be more pronounced (Table 3). One possible explanation for this difference is that skull shape, as a proxy of dietary and life‐mode adaptations (Klaczko et al., 2016; Monteiro et al., 1999; Nogueira et al., 2009), is under strong selection to enhance individual fitness in a given environment (Myers, Lundrigan, Gillespie, & Zelditch, 1996). For example, intraspecific changes in cranial masticatory muscle attachments have been linked to varying levels of insectivory in armadillos as a morphogenetic response to the availability of food resources in heterogeneous habitats (Feijó, 2017). Myers et al. (1996) showed that changing only the consistency of the food (hard and soft diets) was enough to promote intraspecific cranial and dental divergence in rodents. Likewise, Fornel, Cordeiro‐Estrela, and Freitas (2010) found that rodents inhabiting distinct habitats (sand dunes and fields) show significant skull shape differences. Alternatively, while skull shape is a three‐dimensional structure and thus can display diverse phenotypes, size can change only by increasing or decreasing. The dynamic spatial interaction between abiotic and biotic forces along elevational gradients might generate competing pressures (see Table 4) on size changes and thereby minimize the differences between populations across elevation zones (Kawecki & Ebert, 2004; McCain & Grytnes, 2010).

The general change in size as a function of altitude exhibited a nonpositive (negative or neutral) pattern across the nine species. This finding is consistent with that of previous studies on animals along elevational gradients at intraspecific (Caro et al., 2013; Eastman, Morelli, Rowe, Conroy, & Moritz, 2012; Liao, Zhang, & Liu, 2006; Wasserman & Nash, 1979) and interspecific (Du et al., 2017; Geraghty, Dunn, & Sanders, 2007; Hu, Xie, Li, & Jiang, 2011) levels. Conversely, a positive relationship between size and altitude, as expected under Bergmann's rule, has also been reported in the literature (Keller et al., 2013). Such conflicting patterns might reflect idiosyncratic responses due to inherent ecological, physiological, and evolutionary constraints (Adams & Church, 2008; Itescu et al., 2018). Nonetheless, the nonpositive trend shared by moles, shrews, and rodents points to a more common scenario. Sampling part of a species' (altitudinal) range may fail to reveal the real trend, as shown by Adams and Church (2008) when testing Bergmann's rule in salamanders. Based on a large dataset, they found no correlation between body size and temperature in most of the species examined, contrary to previous studies that derived conclusions from limited geographic sampling. Moreover, we expect contrasting patterns depending on the species' elevational range (Figure 3). For example, the size of *A. ilex* decreased with an increase in elevation up to 3,200 m and then exhibited a strong positive relationship with elevation; the size of *N. confucianus* presented a weak negative relationship with increasing elevation up to 2,000 m, followed by a positive relationship at middle and high elevations. These inconsistencies might be linked to dynamic multi‐interactions of selective drivers in each zone.

At high elevations, the reduced species richness (Wen et al., 2017) and predation pressure (Fu et al., 2007; Kumar, Longino, Colwell, & O'Donnell, 2009; this study) may alleviate the effects of biotic interactions on individual size, making climatic factors the main drivers of morphology variation. This hypothesis can explain opposing clines between congeners distributed across distinct altitudinal ranges. For example, the size of the middle‐elevation Daurian pika (*Ochotona daurica*) shows a negative trend as elevation increases (Liao et al., 2006), while that of the high‐altitude plateau pika (*Ochotona curzoniae*) presents a positive trend (Lin, Ci, Zhang, & Su, 2008). Regarding skull shape, we found a lack of consistency in changes across elevation zones, even between sympatric species, such as *Allactaga sibirica* and *Dipus sagitta*, revealing that different phenotypes may be equally effective in similar habitats. This many‐to‐one mapping scenario predicts that complex morphological structures, such as the skull, can perform similar functions with divergent forms (Maestri et al., 2017; Renaud et al., 2018; Wainwright, 2007; Wainwright, Alfaro, Bolnick, & Hulsey, 2005). Alternatively, selection for reduced niche overlap in areas with marked seasonality of resources, such as those at high altitudes, might favor the co‐occurrence of distinct phenotypes.

The ecological parameters associated with phenotypic divergence along elevational gradients are expected to be tied to species ecological and evolutionary traits (Goodyear & Pianka, 2008; Itescu et al., 2018; Keller et al., 2013; Ohsawa & Ide, 2008). Consistent with this expectation, our models revealed somewhat inconsistent responses between shrews and rodents. The reduced exposure of shrews due to their fossorial habits (Healy et al., 2014) and asocial organization (Churchfield, 1990) might explain the reduced influence of biotic interactions on their phenotypic variability. Curiously, the size and shape of the mole *Uropsilus soricipes,* a close relative of shrews that lacks adaptation to fossorial life, were significantly associated with interspecific competition. In addition, phenotypic changes in the two semidesert dwellers (*A. sibirica* and *D. sagitta*) were mainly associated with seasonal temperature and precipitation, while forest dwellers tended to be affected by multiple parameters.

Among the biotic traits, predator richness affected morphological cranial changes the most. While numerous studies have reported the influence of predation risk on community structure (Nelson, Matthews, & Rosenheim, 2004), foraging behavior (Altendorf, Laundré, Gonzalez, & Brown, 2001; Creel, 2011), and habitat use (Creel, Christianson, Liley, & Winnie, 2007; Dickman, 1992; Kotler, Brown, Slotow, Goodfriend, & Strauss, 1993), its effects on morphological variation have been largely overlooked. In mammals, predator richness is traditionally linked to body size variation in insular systems, where relaxed predation is correlated with an increase in size in small species (Heaney, 1978; Michaux et al., 2002). Surprisingly, we found a positive correlation between size and predation risk in most cases (Table 4). This relationship might be an indirect response to reduced prey species abundance at lower altitudes (Fu et al., 2007; Wen et al., 2018), which leads to relaxed competition for food (Heaney, 1978). Notably, our conclusions are derived from a coarse‐grained approach using species' ranges to predict local predator richness. Although our two predator richness parameters generated similar results and agreed with results from previous studies showing higher predation risk at low elevations (Fu et al., 2007), future studies exploring predator–prey interactions along mountain gradients using locally collected data are necessary to test our hypothesis that predation risk is an important factor associated with the intraspecific diversification of the mammalian skull along elevational gradients, a previously neglected parameter.

An open question derived from our study is which main biological factor(s) drives phenotypic divergence at each elevation zone. Answering this question could provide us with a better understanding of the dynamic ecological interactions along altitudinal gradients (Bolnick et al., 2011; Hart, Schreiber, & Levine, 2016; Violle et al., 2012). Because of our limited sample size in each zone, we could not test the effects of our parameters separately. Nevertheless, we anticipate that at lower elevations biotic interactions will play a stronger role in shaping phenotypic changes, whereas at high elevations due to a relaxed predation and lower competition, climatic factors will be the main drivers.

## 5 CONCLUSION

By comparing phylogenetically and ecologically diverse species of rodents, moles, and shrews, we found that phenotypic divergence in mammalian skulls along elevational gradients is a common phenomenon. The underlying causes of this phenomenon are partly related to species' life‐history attributes. Fossorial and solitary animals are mainly affected by climatic factors, while terrestrial and more gregarious species are influenced by biotic and abiotic parameters. Animals living at lower elevations differ greatly in both phenotype and genotype from those living at high elevations, possibly due to the strong divergent selection acting upon populations at opposite ends of elevational gradients. Therefore, moderate selection faced by animals at middle altitudes seems to favor intermediate forms able to exploit distinct elevation zones. Skull size exhibits a nonpositive trend along elevational gradients, although mixed patterns were detected across elevation zones, possibly due to dynamic multi‐interactions among selective drivers. Skull shape displayed contrasting changes between sympatric species across elevational gradients, suggesting equally effective phenotypes in similar habitats. Moreover, we detected consistent population genetic structure mirroring the phenotypic results, which was mainly driven by environmental heterogeneity along mountain slopes (with no or a weak spatial effect), fitting the IBE scenario.

## CONFLICT OF INTEREST

None declared.

## AUTHOR CONTRIBUTIONS

AF conceived the idea; AF, ZW, and QY designed the research; ZW, JC, DG, LX, and QY conducted the field sampling; AF, ZW, JC, and DG curated the data; and AF analyzed the data and led the writing.

## Data Availability

A list of all specimens examined including their museum collection numbers and localities is presented in AppendixS1. The list of predators of small mammals in China compiled in this study with their altitudinal range is presented in AppendixS4. GenBank accession numbers of the cytochrome b sequences of *Niviventer confucianus* used here are listed in AppendixS5.

## References

[ece35273-bib-0001] Adams, D. C. , & Church, J. O. (2008). Amphibians do not follow Bergmann's rule. Evolution, 62, 413–420. 17999723 10.1111/j.1558-5646.2007.00297.x

[ece35273-bib-0002] Adams, D. C. , Collyer, M. L. , Kaliontzopoulou, A. , & Sherratt, E. (2017). Geomorph: Software for geometric morphometric analyses. R package version 3.0.5.

[ece35273-bib-0003] Allendorf, F. W. , & Luikart, G. (2007). Conservation and the genetics of populations. Malden, MA: Blackwell Publishing.

[ece35273-bib-0004] Altendorf, K. B. , Laundré, J. W. , Gonzalez, C. A. L. , & Brown, J. S. (2001). Assessing effects of predation on foraging behavior of mule deer. Journal of Mammalogy, 82, 430–439.

[ece35273-bib-0005] Barton, K. (2018). MuMIn: Multi‐Model Inference. R package version 1.40.4.

[ece35273-bib-0006] Bates, D. , Maechler, M. , Bolker, B. , & Walker, S. (2015). Fitting Linear Mixed‐Effects Models Using lme4. Journal of Statistical Software, 67, 7080–48.

[ece35273-bib-0007] Bellard, C. , Bertelsmeier, C. , Leadley, P. , Thuiller, W. , & Courchamp, F. (2012). Impacts of climate change on the future of biodiversity. Ecology Letters, 15, 365–377. 22257223 10.1111/j.1461-0248.2011.01736.x PMC3880584

[ece35273-bib-0008] Bivand, R. , & Piras, G. (2015). Comparing implementations of estimation methods for spatial econometrics. Journal of Statistical Software, 63, 7080–36.

[ece35273-bib-0009] Bolnick, D. I. , Amarasekare, P. , Araújo, M. S. , Bürger, R. , Levine, J. M. , Novak, M. , … Vasseur, D. A. (2011). Why intraspecific trait variation matters in community ecology. Trends in Ecology & Evolution, 26, 183–192. 21367482 10.1016/j.tree.2011.01.009 PMC3088364

[ece35273-bib-0010] Boutin, S. , & Lane, J. E. (2014). Climate change and mammals: Evolutionary versus plastic responses. Evolutionary Applications, 7, 29–41. 24454546 10.1111/eva.12121 PMC3894896

[ece35273-bib-0011] Branch, C. L. , Jahner, J. P. , Kozlovsky, D. Y. , Parchman, T. L. , & Pravosudov, V. V. (2017). Absence of population structure across elevational gradients despite large phenotypic variation in mountain chickadees (*Poecile gambeli*). Royal Society Open Science, 4, 170057. 28405402 10.1098/rsos.170057 PMC5383859

[ece35273-bib-0012] Brehm, G. , & Fiedler, K. (2004). Bergmann's rule does not apply to geometrid moths along an elevational gradient in an Andean montane rain forest. Global Ecology and Biogeography, 13, 7–14.

[ece35273-bib-0013] Burnham, K. P. , & Anderson, D. R. (2002). Model selection and multimodel inference: A practical information‐theoretic approach. New York, NY: Springer‐Verlag.

[ece35273-bib-0014] Caro, L. M. , Caycedo‐Rosales, P. C. , Bowie, R. C. , Slabbekoorn, H. , & Cadena, C. D. (2013). Ecological speciation along an elevational gradient in a tropical passerine bird? Journal of Evolutionary Biology, 26, 357–374. 23298144 10.1111/jeb.12055

[ece35273-bib-0015] Cheng, J. , Lv, X. , Xia, L. , Ge, D. , Zhang, Q. , Lu, L. , & Yang, Q. (2017). Impact of orogeny and environmental change on genetic divergence and demographic history of *Dipus sagitta* (Dipodoidea, Dipodinae) since the Pliocene in Inland East Asia. Journal of Mammalian Evolution, 26(2), 253–266. 10.1007/s10914-017-9397-6

[ece35273-bib-0016] Chun, Y. , & Griffith, D. A. (2011). Modeling network autocorrelation in space‐time migration flow data: An eigenvector spatial filtering approach. Annals of the Association of American Geographers, 101, 523–536.

[ece35273-bib-0017] Churchfield, S. (1990). The natural history of Shrews. Ithaca, NY: Christopher Helm Ltd.

[ece35273-bib-0018] Creel, S. (2011). Toward a predictive theory of risk effects: Hypotheses for prey attributes and compensatory mortality. Ecology, 92, 2190–2195. 10.1890/11-0327.1 22352157

[ece35273-bib-0019] Creel, S. , Christianson, D. , Liley, S. , & Winnie, J. A. Jr (2007). Predation risk affects reproductive physiology and demography of elk. Science, 315, 960. 17303746 10.1126/science.1135918

[ece35273-bib-0020] R Development Core Team , (2017). R: A language and environment for statistical computing. Vienna, Austria: R Foundation for Statistical Computing.

[ece35273-bib-0021] Dickman, C. R. (1992). Predation and habitat shift in the house mouse, *Mus Domesticus* . Ecology, 73, 313–322.

[ece35273-bib-0022] Diniz‐Filho, J. A. F. , Bini, L. M. , & Hawkins, B. A. (2003). Spatial autocorrelation and red herrings in geographical ecology. Global Ecology and Biogeography, 12, 53–64.

[ece35273-bib-0023] Drake, A. G. , & Klingenberg, C. P. (2008). The pace of morphological change: Historical transformation of skull shape in St Bernard dogs. Proceedings of the Royal Society of London B, 275, 71–76. 10.1098/rspb.2007.1169 PMC2562403 17956847

[ece35273-bib-0024] Du, Y. , Wen, Z. , Zhang, J. , Lv, X. , Cheng, J. , Ge, D. , … Yang, Q. (2017). The roles of environment, space, and phylogeny in determining functional dispersion of rodents (Rodentia) in the Hengduan Mountains, China. Ecology and Evolution, 7, 10941–10951. 29299271 10.1002/ece3.3613 PMC5743695

[ece35273-bib-0025] Dueser, R. D. , & Hallett, J. G. (1980). Competition and habitat selection in a forest‐floor small mammal fauna. Oikos, 35, 293–297.

[ece35273-bib-0026] Eastman, L. M. , Morelli, T. L. , Rowe, K. C. , Conroy, C. J. , & Moritz, C. (2012). Size increase in high elevation ground squirrels over the last century. Global Change Biology, 18, 1499–1508.

[ece35273-bib-0027] Excoffier, L. , & Lischer, H. E. D. (2010). Arlequin suite ver 3.5: A new series of programs to perform population genetics analyses under Linux and Windows. Molecular Ecology Resources, 10, 564–567. 21565059 10.1111/j.1755-0998.2010.02847.x

[ece35273-bib-0028] Excoffier, L. , Smouse, P. E. , & Quattro, J. M. (1992). Analysis of molecular variance inferred from metric distances among DNA haplotypes: Application to human mitochondrial DNA restriction data. Genetics, 131, 479–491. 1644282 10.1093/genetics/131.2.479 PMC1205020

[ece35273-bib-0029] Feijó, A. (2017). Sistemática do Gênero Dasypus Linnaeus, 1758 (Cingulata). Doctoral thesis. João Pessoa, Brazil: Federal University of Paraiba.

[ece35273-bib-0030] Fick, S. E. , & Hijmans, R. J. (2017). WorldClim 2: New 1‐km spatial resolution climate surfaces for global land areas. International Journal of Climatology, 37, 4302–4315.

[ece35273-bib-0031] Fontaneto, D. , Panisi, M. , Mandrioli, M. , Montardi, D. , Pavesi, M. , & Cardini, A. (2017). Estimating the magnitude of morphoscapes: How to measure the morphological component of biodiversity in relation to habitats using geometric morphometrics. The Science of Nature, 104, 55 10.1007/s00114-017-1475-3 28642973

[ece35273-bib-0032] Forester, B. R. , Lasky, J. R. , Wagner, H. H. , & Urban, D. L. (2018). Comparing methods for detecting multilocus adaptation with multivariate genotype‐environment associations. Molecular Ecology, 27, 2215–2233. 29633402 10.1111/mec.14584

[ece35273-bib-0033] Fornel, R. , Cordeiro‐Estrela, P. , & Freitas, T. R. O. (2010). Skull shape and size variation in *Ctenomys minutus* (Rodentia: Ctenomyidae) in geographical, chromosomal polymorphism, and environmental contexts. Biological Journal of the Linnean Society, 101, 705–720.

[ece35273-bib-0034] Fox, C. W. (2018). Towards a mechanistic understanding of global change ecology. Functional Ecology, 32, 1648–1651.

[ece35273-bib-0035] Freeman, B. G. (2017). Little evidence for Bergmann's rule body size clines in passerines along tropical elevational gradients. Journal of Biogeography, 44, 502–510.

[ece35273-bib-0036] Fu, C. , Wang, J. , Pu, Z. , Zhang, S. , Chen, H. , Zhao, B. , … Wu, J. (2007). Elevational gradients of diversity for lizards and snakes in the Hengduan Mountains, China. Biodiversity and Conservation, 16, 707–726.

[ece35273-bib-0037] Garant, D. , Forde, S. E. , & Hendry, A. P. (2007). The multifarious effects of dispersal and gene flow on contemporary adaptation. Functional Ecology, 21, 434–443.

[ece35273-bib-0038] Ge, D. , Lu, L. , Abramov, A. V. , Wen, Z. , Cheng, J. , Xia, L. , … Yang, Q. (2018). Coalescence models reveal the rise of the white‐bellied rat (*Niviventer confucianus*) following the loss of Asian megafauna. Journal of Mammalian Evolution. 10.1007/s10914-018-9428-y

[ece35273-bib-0039] Geraghty, M. J. , Dunn, R. R. , & Sanders, N. J. (2007). Body size, colony size, and range size in ants (Hymenoptera: Formicidae): Are patterns along elevational and latitudinal gradients consistent with Bergmann's Rule? Myrmecological News, 10, 51–58.

[ece35273-bib-0040] Goodyear, S. E. , & Pianka, E. R. (2008). Sympatric ecology of five species of fossorial snakes (Elapidae) in Western Australia. Journal of Herpetology, 42, 279–285.

[ece35273-bib-0041] Grieco, T. M. , & Rizk, O. T. (2010). Cranial shape varies along an elevation gradient in Gambel's white‐footed mouse (*Peromyscus maniculatus gambelii*) in the Grinnell Resurvey Yosemite transect. Journal of Morphology, 271, 897–909. 10.1002/jmor.10839 20623653

[ece35273-bib-0042] Griffith, D. A. (2003). Spatial autocorrelation and spatial filtering: Gaining understanding through theory and scientific visualization. Berlin Heidelberg, Germany: Springer‐Verlag.

[ece35273-bib-0043] Griffith, D. , & Chun, Y. (2014). Spatial autocorrelation and spatial filtering InFischer M. M., & Nijkamp P. (Eds.), Handbook of Regional Science (p. 1732). Berlin Heidelberg, Germany: Springer‐Verlag.

[ece35273-bib-0044] Grueber, C. E. , Nakagawa, S. , Laws, R. J. , & Jamieson, I. G. (2011). Multimodel inference in ecology and evolution: Challenges and solutions. Journal of Evolutionary Biology, 24, 699–711. 21272107 10.1111/j.1420-9101.2010.02210.x

[ece35273-bib-0045] Gutiérrez‐Pinto, N. , McCracken, K. G. , Alza, L. , Tubaro, P. , Kopuchian, C. , Astie, A. , & Cadena, C. D. (2014). The validity of ecogeographical rules is context‐dependent: Testing for Bergmann' and Allen's rules by latitude and elevation in a widespread Andean duck. Biological Journal of the Linnean Society, 111, 850–862.

[ece35273-bib-0046] Harrell, F. E. (2001). Regression modeling strategies: With applications to linear models, logistic regression, and survival analysis (2nd ed.). New York, NY: Springer International Publishing.

[ece35273-bib-0047] Harrison, X. A. , Donaldson, L. , Correa‐Cano, M. E. , Evans, J. , Fisher, D. N. , Goodwin, C. E. D. , … Inger, R. (2018). A brief introduction to mixed effects modelling and multi‐model inference in ecology. PeerJ, 6, e4794. 29844961 10.7717/peerj.4794 PMC5970551

[ece35273-bib-0048] Hart, S. P. , Schreiber, S. J. , & Levine, J. M. (2016). How variation between individuals affects species coexistence. Ecology Letters, 19, 825–838. 27250037 10.1111/ele.12618

[ece35273-bib-0049] He, K. , Hu, N. Q. , Chen, X. , Li, J. T. , & Jiang, X. L. (2016). Interglacial refugia preserved high genetic diversity of the Chinese mole shrew in the mountains of southwest China. Heredity, 116, 23–32. 26286667 10.1038/hdy.2015.62 PMC4675870

[ece35273-bib-0050] Healy, K. , Guillerme, T. , Finlay, S. , Kane, A. , Kelly, S. B. A. , McClean, D. , … Cooper, N. (2014). Ecology and mode‐of‐life explain lifespan variation in birds and mammals. Proceedings of the Royal Society B: Biological Sciences, 281, 20140298–20140298. 10.1098/rspb.2014.0298 PMC4043093 24741018

[ece35273-bib-0051] Heaney, L. R. (1978). Island area and body size of insular mammals: Evidence from the tri‐colored squirrel (*Callosciurus prevosti*) of Southeast Asia. Evolution, 32, 29–44. 28564084 10.1111/j.1558-5646.1978.tb01096.x

[ece35273-bib-0052] Heino, J. , & Gronroos, M. (2014). Untangling the relationships among regional occupancy, species traits, and niche characteristics in stream invertebrates. Ecology and Evolution, 4, 1931–1942. 24963387 10.1002/ece3.1076 PMC4063486

[ece35273-bib-0053] Hijmans, R. J. (2017). raster: geographic data analysis and modeling. R package version 2.6‐7.

[ece35273-bib-0054] Hoyo, J. , Elliott, W. , & Sargatal, J. (1999). Handbook of the birds of the world. (5). Barn-Owls to hummingbirds. Barcelona, Spain: Lynx Edicions.

[ece35273-bib-0055] Hu, J. , Xie, F. , Li, C. , & Jiang, J. (2011). Elevational patterns of species richness, range and body size for spiny frogs. PLoS ONE, 6, e19817. 21611199 10.1371/journal.pone.0019817 PMC3096645

[ece35273-bib-0056] Imhoff, M. L. , & Bounoua, L. (2006). Exploring global patterns of net primary production carbon supply and demand using satellite observations and statistical data. Journal of Geophysical Research, 111, 7080–8.

[ece35273-bib-0057] Imhoff, M. L. , Bounoua, L. , Ricketts, T. , Loucks, C. , Harriss, R. , & Lawrence, W. T. (2004). HANPP Collection: Global Patterns in Net Primary Productivity (NPP). Palisades, NY: NASA Socioeconomic Data and Applications Center (SEDAC).

[ece35273-bib-0058] Itescu, Y. , Schwarz, R. , Donihue, C. M. , Slavenko, A. , Roussos, S. A. , Sagonas, K. , … Meiri, S. (2018). Inconsistent patterns of body size evolution in co‐occurring island reptiles. Global Ecology and Biogeography, 27, 538–550.

[ece35273-bib-0059] IUCN (2017). The IUCN Red List of Threatened Species. Version 2017–3. Retrieved from http://www.iucnredlist.org

[ece35273-bib-0060] Kawecki, T. J. , & Ebert, D. (2004). Conceptual issues in local adaptation. Ecology Letters, 7, 1225–1241.

[ece35273-bib-0061] Keller, I. , Alexander, J. M. , Holderegger, R. , & Edwards, P. J. (2013). Widespread phenotypic and genetic divergence along altitudinal gradients in animals. Journal of Evolutionary Biology, 26, 2527–2543. 24128377 10.1111/jeb.12255

[ece35273-bib-0062] Kierepka, E. M. , & Latch, E. K. (2015). Performance of partial statistics in individual‐based landscape genetics. Molecular Ecology Resources, 15, 512–525. 25230016 10.1111/1755-0998.12332

[ece35273-bib-0063] Klaczko, J. , Sherratt, E. , & Setz, E. Z. F. (2016). Are diet preferences associated to skulls shape diversification in Xenodontine snakes? PLoS ONE, 11, e0148375. 26886549 10.1371/journal.pone.0148375 PMC4757418

[ece35273-bib-0064] Körner, C. (2007). The use of ‘altitude’ in ecological research. Trends in Ecology & Evolution, 22, 569–574. 17988759 10.1016/j.tree.2007.09.006

[ece35273-bib-0065] Kotler, B. P. , Brown, J. S. , Slotow, R. H. , Goodfriend, W. L. , & Strauss, M. (1993). The influence of snakes on the foraging behavior of gerbils. Oikos, 67, 309–316.

[ece35273-bib-0066] Krebs, C. J. (1966). Demographic changes in fluctuating populations of *Microtus californicus* . Ecological Monographs, 36, 239–273.

[ece35273-bib-0067] Krebs, C. J. , Boonstra, R. , Gilbert, S. , Reid, D. , Kenney, A. J. , & Hofer, E. J. (2011). Density estimation for small mammals from livetrapping grids: Rodents in northern Canada. Journal of Mammalogy, 92, 974–981.

[ece35273-bib-0068] Kumar, A. , Longino, J. T. , Colwell, R. K. , & O'Donnell, S. (2009). Elevational patterns of diversity and abundance of Eusocial paper wasps (Vespidae) in Costa Rica. Biotropica, 41, 338–346.

[ece35273-bib-0069] Kumar, S. , Stecher, G. , & Tamura, K. (2016). MEGA7: Molecular Evolutionary Genetics Analysis version 7.0 for bigger datasets. Molecular Biology and Evolution, 33, 1870. 27004904 10.1093/molbev/msw054 PMC8210823

[ece35273-bib-0070] Li, W. H. , & Zhang, Y. G. (2010). Vertical climate of Hengduan Mountains and the impact on forest distribution. Beijing, China: China Meteorological Press.

[ece35273-bib-0071] Liao, J. , Zhang, Z. , & Liu, N. (2006). Altitudinal variation of skull size in Daurian pika (*Ochotona daurica* Pallas, 1868). Acta Zoologica Academiae Scientiarum Hungaricae, 52, 319–329.

[ece35273-bib-0072] Lin, G. , Ci, H. , Zhang, T. , & Su, J. (2008). Conformity of Bergmann's rule in the plateau pika (*Ochotona curzoniae* Hodgson, 1857) on the Qinghai‐Tibetan Plateau. Acta Zoologica Academiae Scientiarum Hungaricae, 54, 411–418.

[ece35273-bib-0073] Liu, R. R. , Ge, D. Y. , Lu, L. , Xia, L. , Liu, Q. Y. , & Yang, Q. S. (2018). Identification and distribution of *Apodemus* species with DNA barcoding in China [in Chinese]. Chinese Journal of Vector Biology and Control, 28, 97–103.

[ece35273-bib-0074] Lv, X. , Cheng, J. , Meng, Y. , Chang, Y. , Xia, L. , Wen, Z. , … Yang, Q. (2018). Disjunct distribution and distinct intraspecific diversification of *Eothenomys melanogaster* in South China. BMC Evolutionary Biology, 18, 50–64. 10.1186/s12862-018-1168-3 29636000 PMC5894153

[ece35273-bib-0075] Ma, J. , Wu, Y.‐J. , Xia, L. , Zhang, Q. , Ma, Y. , & Yang, Q.‐S. (2010). Elevational diversity of small mammals in Luoji Mt. Nature Reserve, Sichuan Province. Acta Theriologica Sinica, 30, 400–410.

[ece35273-bib-0076] Maestri, R. , Fornel, R. , Gonçalves, G. L. , Geise, L. , Freitas, T. R. O. , & Carnaval, A. C. (2016). Predictors of intraspecific morphological variability in a tropical hotspot: Comparing the influence of random and non‐random factors. Journal of Biogeography, 43, 2160–2172. 10.1111/jbi.12815

[ece35273-bib-0077] Maestri, R. , Monteiro, L. R. , Fornel, R. , Upham, N. S. , Patterson, B. D. , & Freitas, T. R. O. (2017). The ecology of a continental evolutionary radiation: Is the radiation of sigmodontine rodents adaptive? Evolution, 71, 610–632. 28025827 10.1111/evo.13155

[ece35273-bib-0078] Martinez, P. A. , Pia, M. V. , Bahechar, I. A. , Molina, W. F. , Bidaum, C. J. , & Montoya‐Burgos, J. I. (2018). The contribution of neutral evolution and adaptive processes in driving phenotypic divergence in a model mammalian species, the Andean fox *Lycalopex culpaeus* . Journal of Biogeography, 45, 7080–12.

[ece35273-bib-0079] McCain, C. M. , & Grytnes, J.‐A. (2010). Elevational gradients in species richness In Encyclopedia of life sciences (pp. 7080 – 10). Chichester, UK: John Wiley & Sons, Ltd.

[ece35273-bib-0080] Merilä, J. , & Hendry, A. P. (2014). Climate change, adaptation, and phenotypic plasticity: The problem and the evidence. Evolutionary Applications, 7, 7080–14. 10.1111/eva.12137 PMC3894893 24454544

[ece35273-bib-0081] Michaux, J. R. , Bellocq, J. G. , Sarà, M. , & Morand, S. (2002). Body size increase in insular rodent populations: A role for predators? Global Ecology and Biogeography, 11, 427–436. 10.1046/j.1466-822x.2002.00301.x

[ece35273-bib-0082] Mitteroecker, P. , & Bookstein, F. (2011). Linear discrimination, ordination, and the visualization of selection gradients in modern morphometrics. Evolutionary Biology, 38, 100–114.

[ece35273-bib-0083] Mitteroecker, P. , Gunz, P. , Windhager, S. , & Schaefer, K. (2013). A brief review of shape, form, and allometry in geometric morphometrics, with applications to human facial morphology. Hystrix, 24, 59–66.

[ece35273-bib-0084] Monteiro, L. R. , Lessa, L. G. , & Are, A. S. (1999). Ontogenetic variation in skull shape of *Thrichomys apereoides* (Rodentia, Echimyidae). Journal of Mammalogy, 80, 102–111.

[ece35273-bib-0085] Myers, P. , Lundrigan, B. L. , Gillespie, B. W. , & Zelditch, M. L. (1996). Phenotypic plasticity in skull and dental morphology in the prairie deer mouse (*Peromyscus maniculatus bairdii*). Journal of Morphology, 229, 229–237. 8755340 10.1002/(SICI)1097-4687(199608)229:2<229::AID-JMOR7>3.0.CO;2-W

[ece35273-bib-0086] Nelson, E. H. , Matthews, C. E. , & Rosenheim, J. A. (2004). Predators reduce prey population growth by inducing changes in prey behavior. Ecology and Evolution, 85, 1853–1858.

[ece35273-bib-0087] Nogueira, M. R. , Peracchi, A. L. , & Monteiro, L. R. (2009). Morphological correlated of bite force and diet in the skull and mandible of phyllostomid bats. Functional Ecology, 23, 715–723.

[ece35273-bib-0088] Nosil, P. , Harmon, L. J. , & Seehausen, O. (2008). Ecological explanations for (incomplete) speciation. Trends in Ecology & Evolution, 24, 145–156. 10.1016/j.tree.2008.10.011 19185951

[ece35273-bib-0089] Ohsawa, M. (1991). Structural comparison of tropical montane rain forests along latitudinal and altitudinal gradients in south and east Asia. Vegetatio, 97, 7080–10.

[ece35273-bib-0090] Ohsawa, M. (1993). Latitudinal pattern of mountain vegetation zonation in southern and eastern Asia. Journal of Vegetation Science, 4, 13–18.

[ece35273-bib-0091] Ohsawa, T. , & Ide, Y. (2008). Global patterns of genetic variation in plant species along vertical and horizontal gradients on mountains. Global Ecology and Biogeography, 17, 152–163.

[ece35273-bib-0092] Oksanen, J. , Blanchet, G. F. , Friendly, M. , Kindt, R. , Legendre, P. , McGlinn, D. , Wagner, E. (2018). vegan: Community Ecology Package. R package version 2.4‐6.

[ece35273-bib-0093] Pacheco, M. , Kajin, M. , GentileI, R. , Zangrandi, P. L. , Vieira, M. V. , & Cerqueira, R. (2013). A comparison of abundance estimators for small mammal populations. Zoologia, 30, 182–190.

[ece35273-bib-0094] Paterson, S. (2003). Mixed models: Getting the best use of parasitological data. Trends in Parasitology, 19, 370–375. 12901939 10.1016/s1471-4922(03)00149-1

[ece35273-bib-0095] Perez, S. I. , Diniz‐Filho, J. A. F. , Bernal, V. , & Gonzalez, P. N. (2010). Spatial regression techniques for inter‐population data: Studying the relationships between morphological and environmental. Journal of Evolutionary Biology, 23, 237–248. 20002248 10.1111/j.1420-9101.2009.01905.x

[ece35273-bib-0096] Renaud, S. , Ledevin, R. , Pisanu, B. , Chapuis, J. L. , Quillfeldt, P. , & Hardouin, E. A. (2018). Divergent in shape and convergent in function: Adaptive evolution of the mandible in Sub‐Antarctic mice. Evolution, 72, 878–892. 29528493 10.1111/evo.13467

[ece35273-bib-0097] Richards, S. A. (2008). Dealing with overdispersed count data in applied ecology. Journal of Applied Ecology, 45, 218–227.

[ece35273-bib-0098] Rohlf, F. J. (2017). tpsDig, version 2.3. Department of Ecology and Evolution, State University of New York, Stony Brook.

[ece35273-bib-0099] Rosenblum, E. B. (2006). Convergent evolution and divergent selection: Lizards at the white sands ecotone. The American Naturalist, 167, 7080–15. 10.1086/498397 16475095

[ece35273-bib-0100] Rozas, J. , Ferrer‐Mata, A. , Sánchez‐DelBarrio, J. C. , Guirao‐Rico, S. , Librado, P. , Ramos‐Onsins, S. E. , & Sánchez‐Gracia, A. (2017). DnaSP v6: DNA sequence polymorphism analysis of large datasets. Molecular Biology and Evolution, 34, 3299–3302. 29029172 10.1093/molbev/msx248

[ece35273-bib-0101] Schielzeth, H. , & Forstmeier, W. (2008). Conclusions beyond support: Overconfident estimates in mixed models. Behavioral Ecology, 20, 416–420. 19461866 10.1093/beheco/arn145 PMC2657178

[ece35273-bib-0102] Schlager, S. (2017). Morpho and Rvcg – Shape analysis in R In Zheng G., Li S., & Szekely G.(Eds.), Statistical Shape and Deformation Analysis(pp. 217–256). London, UK: Academic Press.

[ece35273-bib-0103] Sexton, J. P. , Hangartner, S. B. , & Hoffmann, A. A. (2014). Genetic isolation by environment or distance: Which pattern of gene flow is most common? Evolution, 68, 7080–15. 10.1111/evo.12258 24111567

[ece35273-bib-0104] Shen, L. , Bao, Y. , Xu, Z. , Wei, D. , & Liu, J. (2011). Effect of different seasons and sex of *Niviventer confucianus* on islands at Thousand Island Lake. Journal of Zhejiang Normal University, 34, 328–332.

[ece35273-bib-0105] Shi, X. , Hu, Q. , Li, J. , Tang, Z. , Yang, J. , Li, W. , … Li, S. (2017). Camera‐trapping surveys of the mammal and bird diversity in Wolong National Nature Reserve, Sichuan Province [in Chinese]. Biodiversity Science, 25, 1131–1136.

[ece35273-bib-0106] Smith, A. T. , & Xie, Y. (2013). Mammals of China. Princeton, NY and Oxford, UK: Princeton University Press.

[ece35273-bib-0107] Tang, C. Q. , & Ohsawa, M. (1997). Zonal transition of evergreen, deciduous, and coniferous forests along the altitudinal gradient on a humid subtropical mountain, Mt. Emei, Sichuan, China. Plant Ecology, 133, 63–78.

[ece35273-bib-0108] Violle, C. , Enquist, B. J. , McGill, B. J. , Jiang, L. , Albert, C. H. , Hulshof, C. , & Messier, J. (2012). The return of the variance: intraspecific variability in community ecology. Trends in Ecology and Evolution, 27, 244–252. 22244797 10.1016/j.tree.2011.11.014

[ece35273-bib-0109] Wainwright, P. C. (2007). Functional versus morphological diversity in macroevolution. Annual Review of Ecology, Evolution, and Systematics, 38, 381–401.

[ece35273-bib-0110] Wainwright, P. C. , Alfaro, M. E. , Bolnick, D. I. , & Hulsey, C. D. (2005). Many‐to‐one mapping of form to function: A general principle in organismal design? Integrative and Comparative Biology, 45, 256–262. 21676769 10.1093/icb/45.2.256

[ece35273-bib-0111] Wang, I. J. , & Bradburd, G. S. (2014). Isolation by environment. Molecular Ecology, 23, 5649–5662. 25256562 10.1111/mec.12938

[ece35273-bib-0112] Wang, I. J. , & Summers, K. (2010). Genetic structure is correlated with phenotypic divergence rather than geographic isolation in the highly polymorphic strawberry poison‐dart frog. Molecular Ecology, 19, 447–458. 20025652 10.1111/j.1365-294X.2009.04465.x

[ece35273-bib-0113] Wasserman, D. , & Nash, D. J. (1979). Variation in body size, hair length in Peromyscus along an altitudinal gradient. Holarctic Ecology, 2, 115–118.

[ece35273-bib-0114] Waterhouse, M. D. , Erb, L. P. , Beever, E. A. , & Russello, M. A. (2018). Adaptive population divergence and directional gene flow across steep elevational gradients in a climate‐sensitive mammal. Molecular Ecology, 27, 2512–2528. 29693300 10.1111/mec.14701

[ece35273-bib-0115] Wen, Z. , Wu, Y. , Cheng, J. , Cai, T. , Du, Y. , Ge, D. , Yang, Q. (2018). Abundance of small mammals correlates with their elevational range sizes and elevational distributions in the subtropics. Ecography, 41(11), 1888–1898. 10.1111/ecog.03558.

[ece35273-bib-0116] Wen, Z. , Wu, Y. I. , Ge, D. , Cheng, J. , Chang, Y. , Yang, Z. , … Yang, Q. (2017). Heterogeneous distributional responses to climate warming: Evidence from rodents along a subtropical elevational gradient. BMC Ecology, 17, 7080–9. 10.1186/s12898-017-0128-x PMC5397755 28427386

[ece35273-bib-0117] Wu, Y. , Yang, Q. , Wen, Z. , Xia, L. , Zhang, Q. , & Zhou, H. (2013). What drives the species richness patterns of non‐volant small mammals along a subtropical elevational gradient? Ecography, 36, 185–196.

[ece35273-bib-0118] Zhong, X. , Zhang, W. , & Luo, J. (1999). The characteristics of the mountain ecosystem and environment in the Gongga Mountain region. Ambio, 28, 648–654.

