# Supplementary material for: Divergent selection along elevational gradients promotes genetic and phenotypic disparities among small mammal populations
Source: Ecol Evol. 2019 May 28;9(12):7080–95. doi: 10.1002/ece3.5273 (PMC6662404; doi:10.1002/ece3.5273)
Supplement: Supplementary file 1 [file ECE3-9-7080-s001.docx]

**Divergent selection along elevational gradients promotes genetic and phenotypic disparities among small mammal populations**

**Supplementary Files**

**Appendix S1.** List of specimens examined in the present study with their collection localities and altitude (in metres). Specimens are deposited in the mammal collection of the Institute of Zoology, Chinese Academy of Science, Beijing, China.

*Anourosorex squamipes* (73 specimens). **Sichuan:** Xichang city, Luoji Mountain: 2590m (IOZCAS-BBD085); 2279m (IOZCAS-SWS036, IOZCAS-SWS060). Guangyuan city, Tangjiahe Nature reserve: 2382m (IOZCAS-HHC369, IOZCAS-HHC359;1863m (IOZCAS-HTA016, IOZCAS-HTA022, IOZCAS-HTC322, IOZCAS-HTD312, IOZCAS-HYC125, IOZCAS-HYC331, IOZCAS-HYC332, IOZCAS-HYC344, IOZCAS-HYC353, IOZCAS-HYD124, IOZCAS-HYD354); 2837m (IOZCAS-KKD259, IOZCAS-KKC223); 2382m (IOZCAS-YCC393, IOZCAS-YCC394, IOZCAS-YCC395, IOZCAS-YCC406, IOZCAS-YCC407, IOZCAS-YCC412, IOZCAS-YCD397). Wenchuan County, Wolong Nature reserve: 2029m (IOZCAS-WL140006, IOZCAS-WL140043, IOZCAS-WL140044, IOZCAS-WL140045, IOZCAS-WL140054, IOZCAS-WL140055, IOZCAS-WL140057, IOZCAS-WL140063); 1973m (IOZCAS-WL140196, IOZCAS-WL140197, IOZCAS-WL140206, IOZCAS-WL140227, IOZCAS-WL140250, IOZCAS-WL140253); 2463m (IOZCAS-WL140280, IOZCAS-WL140297, IOZCAS-WL140298, IOZCAS-WL140301); 1816m (IOZCAS-WL140330, IOZCAS-WL140333, IOZCAS-WL140341); 1123m (IOZCAS-WL16001, IOZCAS-WL16002); 1729m (IOZCAS-WL16014, IOZCAS-WL16032, IOZCAS-WL16033, IOZCAS-WL16034, IOZCAS-WL16036, IOZCAS-WL16086, IOZCAS-WL16112, IOZCAS-WL16118, IOZCAS-WL16154, IOZCAS-WL16160, IOZCAS-WL16174, IOZCAS-WL16181, IOZCAS-WL16201, IOZCAS-WL16219, IOZCAS-WL16228, IOZCAS-WL16229, IOZCAS-WL16233, IOZCAS-WL16250, IOZCAS-WL16263, IOZCAS-WL16276, IOZCAS-WL16279, IOZCAS-WL16282); 2006m (IOZCAS-WL16435, IOZCAS-WL16447, IOZCAS-WL16451).

*Soriculus nigrescens* (32 specimens). **Tibet**: Linzhi: 3332m (IOZCAS-CG20140149B); 2760m (IOZCAS-DJ20140048, IOZCAS-DJ20140575, IOZCAS-DJ20140603, IOZCAS-DJ20140630, IOZCAS-DJ20140631, IOZCAS-DJ20140632, IOZCAS-DJ20140633, IOZCAS-DJ20140657); 4194m (IOZCAS-DY20140340); 3332m (IOZCAS-GC20140125, IOZCAS-GC20140130, IOZCAS-GC20140137, IOZCAS-GC20140146, IOZCAS-GC20140162, IOZCAS-GC20140163, IOZCAS-GC20140179, IOZCAS-GC20140180); 3381m (IOZCAS-GC20140435); 3009m (IOZCAS-JC20140202, IOZCAS-JC20140203, IOZCAS-JC20140215, IOZCAS-JC20140216); 3203m (IOZCAS-JC20140486, IOZCAS-JC20140499, IOZCAS-JC20140500); 3891m (IOZCAS-ST20140262, IOZCAS-ST20140305); 3556m (IOZCAS-ZD20140220, IOZCAS-ZD20140237); 3767m (IOZCAS-ZD20140402, IOZCAS-ZD20140411).

*Uropsilus soricipes* (26 specimens). **Sichuan:** Tibetan Autonomous Prefecture of Garzê, Gongga Mountain: 2878m (IOZCAS-HB20277, IOZCAS-HB20299); 2099m (IOZCAS-YB10076). Guangyuan city, Tangjiahe Nature reserve: 2382m (IOZCAS-HHC367, IOZCAS-HHC368, IOZCAS-HHC376, IOZCAS-HHD372, IOZCAS-YCC392, IOZCAS-YCC409, IOZCAS-YCC410, IOZCAS-YCD399); 1863m (IOZCAS-HTB011, IOZCAS-HTC319, IOZCAS-HTD115, IOZCAS-HTD304, IOZCAS-HTD327, IOZCAS-HYC329, IOZCAS-HYD339, IOZCAS-HYD349); 2837m (IOZCAS-KKC150, IOZCAS-KKD208, IOZCAS-KKD230). Wenchuan County, Wolong Nature reserve: 1973m (IOZCAS-WL140210); 1729m (IOZCAS-WL16075); 1340m (IOZCAS-WL15363). Tibetan Qiang Autonomous Prefecture of Ngawa: 1929m (IOZCAS-WL15433).

*Eothenomys melanogaster* (26 specimens). **Sichuan**: Guangyuan city, Tangjiahe Nature reserve: 1863m (IOZCAS-HTC109, IOZCAS-HTC309, IOZCAS-HTD302, IOZCAS-HYB032); 1221m (IOZCAS-JCC287, IOZCAS-JCD284). Wenchuan County, Wolong Nature reserve: 1944m (IOZCAS-WL15145, IOZCAS-WL15270); 1729m (IOZCAS-WL16047, IOZCAS-WL16124, IOZCAS-WL16132, IOZCAS-WL16256). Zhejiang, Yiwu city: 103m (IOZCAS-YW14002, IOZCAS-YW14003, IOZCAS-YW14004, IOZCAS-YW14005, IOZCAS-YW14006, IOZCAS-YW14007, IOZCAS-YW14009, IOZCAS-YW14010, IOZCAS-YW14011, IOZCAS-YW14012, IOZCAS-YW14013, IOZCAS-YW14014, IOZCAS-YW14015). **Hubei**: Qingfengcun: 1410m (IOZCAS-PQ026).

*Allactaga sibirica* (91 specimens). **Qinghai:** Delingha city: 2881m (IOZCAS-DL08014, IOZCAS-DL08016, IOZCAS-DL08049, IOZCAS-DL08050, IOZCAS-DL08057, IOZCAS-DL08058, IOZCAS-DL08066, IOZCAS-DL08096, IOZCAS-DL08112, IOZCAS-DL08123, IOZCAS-DL08136, IOZCAS-DL08140). **Gansu:** Jiuquan city: 1170m (IOZCAS-GS08020). 1737m (IOZCAS-GS08069). **Jilin**: Baicheng city: 166m (IOZCAS-JL15010, IOZCAS-JL15016, IOZCAS-JL15018, IOZCAS-JL15019, IOZCAS-JL15020, IOZCAS-JL15021, IOZCAS-JL15022, IOZCAS-JL15023, IOZCAS-JL15026, IOZCAS-JL15027). **Inner Mongolia:** Chifeng city: 697m (IOZCAS-NM15047, IOZCAS-NM15048, IOZCAS-NM15050); 241m (IOZCAS-NM15051, IOZCAS-NM15052, IOZCAS-NM15055, IOZCAS-NM15059); 697m (IOZCAS-NM15071, IOZCAS-NM15077, IOZCAS-NM15081, IOZCAS-NM15082, IOZCAS-NM15084, IOZCAS-NM15085); 1316m (IOZCAS-NM15116). **Qinghai:** Delingha city: 2970m (IOZCAS-QZ0196, IOZCAS-QZ0197); 2881m (IOZCAS-QZ0216, IOZCAS-QZ0283, IOZCAS-QZ0313, IOZCAS-QZ0316, IOZCAS-QZ0337); 2970m (IOZCAS-QZ0339, IOZCAS-QZ0342); 1311m (IOZCAS-SW003, IOZCAS-SW013, IOZCAS-SW014, IOZCAS-SW016, IOZCAS-SW023, IOZCAS-SW042, IOZCAS-SW092, IOZCAS-SW107, IOZCAS-SW108, IOZCAS-SW109, IOZCAS-SW110, IOZCAS-SW119, IOZCAS-SW122, IOZCAS-SW124, IOZCAS-SW125, IOZCAS-SW126, IOZCAS-SW127, IOZCAS-SW128, IOZCAS-SW133, IOZCAS-SW135, IOZCAS-SW136, IOZCAS-SW137, IOZCAS-SW138, IOZCAS-SW140, IOZCAS-SW141, IOZCAS-SW142, IOZCAS-SW143). **Xinjiang:** Fuyun County: 906m (IOZCAS-XJ09104, IOZCAS-XJ09105, IOZCAS-XJ09107). Beitun County: 564m (IOZCAS-XJ09203, IOZCAS-XJ09205); 555m (IOZCAS-XJ09220). Balikun County: 1740m (IOZCAS-XJ09306, IOZCAS-XJ09307, IOZCAS-XJ09310, IOZCAS-XJ09311); 1822m (IOZCAS-XJ15003). Kelamayi: 299m (IOZCAS-XJ15020). Qinghe county: 1246m (IOZCAS-XJ09066, IOZCAS-XJ09067, IOZCAS-XJ09068, IOZCAS-XJ09074).

*Dipus sagitta* (80 specimens). **Inner Mongolia**: Bayanhaote: 1393m (IOZCAS-BY09023, IOZCAS-BY09025, IOZCAS-BY09028, IOZCAS-BY09029, IOZCAS-BY09030, IOZCAS-BY09031, IOZCAS-BY09032, IOZCAS-BY09035, IOZCAS-BY09036, IOZCAS-BY09037, IOZCAS-BY09038, IOZCAS-BY09039, IOZCAS-BY09040, IOZCAS-BY09041, IOZCAS-BY09042). Horqin Right Wing Middle Banner: 338m (IOZCAS-JL15036, IOZCAS-JL15039). Tongliao city: 241m (IOZCAS-NM15070). Wushen County: 1311m (IOZCAS-SW010, IOZCAS-SW011, IOZCAS-SW017, IOZCAS-SW019, IOZCAS-SW021, IOZCAS-SW034, IOZCAS-SW035, IOZCAS-SW040, IOZCAS-SW060, IOZCAS-SW067, IOZCAS-SW068, IOZCAS-SW071, IOZCAS-SW073, IOZCAS-SW074, IOZCAS-SW079, IOZCAS-SW085, IOZCAS-SW089, IOZCAS-SW095, IOZCAS-SW099, IOZCAS-SW101, IOZCAS-SW106, IOZCAS-SW117, IOZCAS-SW121, IOZCAS-SW129, IOZCAS-SW131). **Qinghai**: Dachaidan county: 3183m (IOZCAS-DC08162, IOZCAS-DC08163, IOZCAS-DC08164, IOZCAS-DC08167, IOZCAS-DC08180, IOZCAS-DC08182). Delingha city: 2881m (IOZCAS-DL08020, IOZCAS-DL08021, IOZCAS-DL08039, IOZCAS-DL08062, IOZCAS-DL08075, IOZCAS-DL08100, IOZCAS-DL08130, IOZCAS-DL08131). Delingha city: 2970m (IOZCAS-QZ0192, IOZCAS-QZ0202, IOZCAS-QZ0338); 2881m (IOZCAS-QZ0252, IOZCAS-QZ0275, IOZCAS-QZ0277, IOZCAS-QZ0285, IOZCAS-QZ0289, IOZCAS-QZ0291, IOZCAS-QZ0308, IOZCAS-QZ0309, IOZCAS-QZ0310, IOZCAS-QZ0311, IOZCAS-QZ0320, IOZCAS-QZ0321). **Gansu:** Jiayuguan: 1737m (IOZCAS-GS08025, IOZCAS-GS08036, IOZCAS-GS08064, IOZCAS-GS8033). **Xinjiang:** Hami city: 971m (IOZCAS-XJ08100, IOZCAS-XJ08101, IOZCAS-XJ08103, IOZCAS-XJ08104).

*Apodemus chevrieri* (35 specimens). **Sichuan:** Xichang city, Luoji Mountain: 2590m (IOZCAS-BB063, IOZCAS-BBD029, IOZCAS-BBD030, IOZCAS-BBD032, IOZCAS-BBD036, IOZCAS-BBD038); 3430m (IOZCAS-AZ021); 1927m (IOZCAS-TMG032). Tibetan Autonomous Prefecture of Garzê, Gongga Mountain: 1487m (IOZCAS-DB10028); 1210m (IOZCAS-MA10006, IOZCAS-MA20016); 3556m (IOZCAS-ZD20140232). Guangyuan city, Tangjiahe Nature reserve: 1221m (IOZCAS-JCC279). Wenchuan County, Wolong Nature reserve 2641m (IOZCAS-WL140153); 2463m (IOZCAS-WL140312); 2006m (IOZCAS-WL16432). **Tibet:** Linzhi: 2760m (IOZCAS-DJ20140049, IOZCAS-DJ20140059, IOZCAS-DJ2014006, IOZCAS-DJ20140099); 3009m (IOZCAS-JC20140197); 2472m (IOZCAS-LY20140031). 2402m (IOZCAS-LY2014681). **Yunnan**: Dali Bai Autonomous Prefecture, Cang Mountain: 3317m (IOZCAS-DL053, IOZCAS-DL088). Jingdong Yi Autonomous County, Ailao Mountain: 2513m (IOZCAS-JD098). Diqing Tibetan Autonomous Prefecture, Baima Mountain: 2584m (IOZCAS-NMB008, IOZCAS-NMB009, IOZCAS-NMB025, IOZCAS-NMC676, IOZCAS-NMD666, IOZCAS-NMD689, IOZCAS-NMD708). Zhaotong City: 3050m (IOZCAS-ZT001). **Guizhou:** Tongren city, Fanjing Mountain: 2009m (IOZCAS-GZ-MM140063).

*Apodemus ilex* (75 specimens). **Yunnan:** Diqing Tibetan Autonomous Prefecture, Baima Mountain: 3368m (IOZCAS-BZA133, IOZCAS-BZB144, IOZCAS-BZB171); 3149m (IOZCAS-AYA084, IOZCAS-AYA085, IOZCAS-AYA099, IOZCAS-AYA105, IOZCAS-AYA112, IOZCAS-AYA115, IOZCAS-AYB088, IOZCAS-AYB120, IOZCAS-AYC506); 3764m (IOZCAS-YKA190, IOZCAS-YKB186); 2584m (IOZCAS-NMA018, IOZCAS-NMA032, IOZCAS-NMA033, IOZCAS-NMA630); 2966m (IOZCAS-JHA053, IOZCAS-JHB040, IOZCAS-JHB078, IOZCAS-JHB081, IOZCAS-JHC565). Jingdong Yi Autonomous County, Ailao Mountain: 2513m (IOZCAS-JD103). Lijiang city, Yulong Mountain: 3326m (IOZCAS-LJ024). **Sichuan:** Xichang city, Luoji Mountain: 2590m (IOZCAS-BBD041). **Tibet:** Linzhi: 2760m (IOZCAS-DJ20140046, IOZCAS-DJ20140054, IOZCAS-DJ20140056, IOZCAS-DJ20140060, IOZCAS-DJ20140061, IOZCAS-DJ20140066, IOZCAS-DJ20140070, IOZCAS-DJ20140078, IOZCAS-DJ20140087, IOZCAS-DJ20140092, IOZCAS-DJ20140098, IOZCAS-DJ20140102, IOZCAS-DJ20140561, IOZCAS-DJ20140562, IOZCAS-DJ20140564, IOZCAS-DJ20140565, IOZCAS-DJ20140586, IOZCAS-DJ20140607, IOZCAS-DJ20140609, IOZCAS-DJ20140610, IOZCAS-DJ20140622, IOZCAS-DJ20140627, IOZCAS-DJ20140666); 3009m (IOZCAS-JC20140187, IOZCAS-JC20140190, IOZCAS-JC20140191, IOZCAS-JC20140192, IOZCAS-JC20140199, IOZCAS-JC20140209, IOZCAS-JC20140212, IOZCAS-JC20140213); 2472m (IOZCAS-LY20140025, IOZCAS-LY20140038, IOZCAS-LY20140039); 3332m (IOZCAS-GC20140164, IOZCAS-GC20140171, IOZCAS-GC20140172); 3981m (IOZCAS-ST20140296); 3556m (IOZCAS-ZD20140221, IOZCAS-ZD20140223, IOZCAS-ZD20140224, IOZCAS-ZD20140225, IOZCAS-ZD20140233, IOZCAS-ZD20140241, IOZCAS-ZD20140246); 2985m (IOZCAS-14ML002); 2745m (IOZCAS-14ZM003, IOZCAS-14ZM004, IOZCAS-14ZM006).

*Niviventer confucianus* (56 specimens). **Sichuan:** Xichang city, Luoji Mountain: 2590m (IOZCAS-BBD047, IOZCAS-BBD050, IOZCAS-BBD057); 3109m (IOZCAS-LZA008, IOZCAS-LZA025, IOZCAS-LZA043, IOZCAS-LZA074); 3430m (IOZCAS-AZ016, IOZCAS-AZ017, IOZCAS-AZ073); 2279m (IOZCAS-SWS037). Tibetan Autonomous Prefecture of Garzê, Gongga Mountain: 1487m (IOZCAS-DA10015, IOZCAS-DB10022, IOZCAS-DB10025, IOZCAS-DB10038, IOZCAS-DB10039, IOZCAS-DB20026, IOZCAS-DB20033, IOZCAS-DB20048); 2878m (IOZCAS-HA10227, IOZCAS-HA10231, IOZCAS-HA10239); 1210m (IOZCAS-MA20004, IOZCAS-MA20010, IOZCAS-MB20001, IOZCAS-MB20002, IOZCAS-MB20013); 2512m (IOZCAS-PA10093, IOZCAS-PA10119, IOZCAS-PA20135); 2099m (IOZCAS-YA10146, IOZCAS-YA10147, IOZCAS-YA10160, IOZCAS-YA10172, IOZCAS-YA10176, IOZCAS-YA20070, IOZCAS-YA20071, IOZCAS-YB10042, IOZCAS-YB10050, IOZCAS-YB10063, IOZCAS-YB10070, IOZCAS-YB10085, IOZCAS-YB20063, IOZCAS-YB20074, IOZCAS-YB20090, IOZCAS-YB20091). Guangyuan city, Tangjiahe Nature reserve: 1863m (IOZCAS-HTC110, IOZCAS-HYC123, IOZCAS-HYC136, IOZCAS-HYD350). Wenchuan County, Wolong Nature reserve: 1973m (IOZCAS-WL140243); 2463m (IOZCAS-WL140294). **Yunnan:** Diqing Tibetan Autonomous Prefecture, Baima Mountain: 2966m (IOZCAS-JHB072); 1584m (IOZCAS-NMD704). **Hubei**: Qingfengcun: 2071m (IOZCAS-YYH014, IOZCAS-YYH016).

**Table S2.** Definition of landmarks (L) in the ventral view of the skull used in this study.

| Eulipotyphla, Soricidae, *Anourosorex* | |
| --- | --- |
| L 1 | Anteriormost medial point of the palate |
| L 2 | Posteriormost medial point of the palate |
| L 3 | Anteriormost medial point of the foramen magnum |
| L 4 | Posteriormost medial point of the foramen magnum |
| L 5 | Lateralmost point of the foramen magnum, aligned with L4 |
| L 6 | Lateralmost point of the braincase, aligned with tympanic ring |
| L 7 | Posteriormost point of the superior articular facet |
| L 8 | Anteriormost point of the superior articular facet |
| L 9 | Epicondylar/postglenoid notch |
| L 10 | Posteriormost point of the inferior articular facets |
| L 11 | Posteriormost point of the last upper molar (M3) |
| L 12 | Lingualmost point of the M2 |
| L 13 | Labialmost point of the M2 |
| L 14 | Lingualmost point of the protocone of the M2 |
| L 15 | Labialmost point of the M2 |
| L 16 | Lingualmost point of the protocone of the M1 |
| L 17 | Labialmost point of the canine |
| L 18 | Lingualmost medial (inner concave) point of the first incisor |
| L 19 | Labialmost medial point of the first incisor |
| L 20 | Anteriormost point of the first incisor |

| Eulipotyphla, Soricidae, *Soriculus* | |
| --- | --- |
| L 1 | Anteriormost medial point of the palate |
| L 2 | Posteriormost medial point of the palate |
| L 3 | Anteriormost medial point of the foramen magnum |
| L 4 | Posteriormost medial point of the foramen magnum |
| L 5 | Lateralmost point of the foramen magnum, aligned with L4 |
| L 6 | Lateralmost point of the braincase, aligned with tympanic ring |
| L 7 | Anteriormost point of the superior articular facet |
| L 8 | Posteriormost point of the last upper molar (M3) |
| L 9 | Lingualmost point of the hypocone of the M2 |
| L 10 | Labialmost point of the M2 |
| L 11 | Lingualmost point of the hypocone of the M1 |
| L 12 | Labialmost point of the M1 |
| L 13 | Lingualmost point of the hypocone of the P1 |
| L 14 | Lingualmost point of the I3 |
| L 15 | Lingualmost medial (inner concave) point of the first incisor |
| L 16 | Labialmost medial point of the first incisor |

| Eulipotyphla, Talpidae, *Uropsilus* | |
| --- | --- |
| L 1 | Anteriormost medial point of the premaxilla |
| L 2 | Posteriormost medial point of the palate |
| L 3 | Anteriormost medial point of the foramen magnum |
| L 4 | Posteriormost medial point of the foramen magnum |
| L 5 | Lateralmost point of the foramen magnum (inner border of occipital condyle) |
| L 6 | Lateralmost point of the braincase, aligned with tympanic ring |
| L 7 | Lateralmost point of the posterior border of the palate |
| L 8 | Posterior inner point of internal orbit |
| L 9 | Posterior lateral point of internal orbit |
| L 10 | Anterior point of internal orbit |
| L 11 | Posteriormost point of the last upper molar (M3) |
| L 12 | Anteriomost point of the P2 |
| L 13 | Posteriormost point of the P1 |
| L 14 | Anteriomost point of the I2 |
| L 15 | Posteriormost point of the I1 |

| Rodentia, Cricetidae, *Eothenomys* | |
| --- | --- |
| L 1 | Anteriormost medial point of the premaxilla |
| L 2 | Anteriormost medial point of the incisive foramen |
| L 3 | Posteriormost medial point of the incisive foramen |
| L 4 | Posteriormost medial point of the palate |
| L 5 | Posteriormost medial point of the foramen magnum |
| L 6 | Lateralmost point of the foramen magnum, aligned with L5 |
| L 7 | Exterior tip of external auditory meatus |
| L 8 | Innermost point of the auditory bullae |
| L 9 | Posterior point of external orbit |
| L 10 | Anterior point of upper maxillary process |
| L 11 | Anterior point of internal orbit |
| L 12 | Lateralmost point of the incisive |
| L 13 | Posteriormost point of the toothrow |
| L 14 | Anteriormost point of the toothrow |

| Rodentia, Dipodidae, *Allactaga* and *Dipus* | |
| --- | --- |
| L 1 | Anteriormost medial point of the premaxilla |
| L 2 | Anteriormost medial point of the incisive foramen |
| L 3 | Posteriormost medial point of the incisive foramen |
| L 4 | Posteriormost medial point of the palate |
| L 5 | Anteriormost medial point of the foramen magnum |
| L 6 | Posteriormost medial point of the foramen magnum |
| L 7 | Exterior tip of external auditory meatus |
| L 8 | Innermost point of the auditory bullae |
| L 9 | Anteriormost point of the auditory bullae |
| L 10 | Posteriormost point of the auditory bullae |
| L 11 | Posterior point of internal orbit |
| L 12 | Anterior point of internal orbit |
| L 13 | Lateralmost point of the incisive |
| L 14 | Posteriormost point of the toothrow |
| L 15 | Anteriormost point of the toothrow |

| Rodentia, Muridae, *Apodemus* | |
| --- | --- |
| L 1 | Anteriormost medial point of the nasal |
| L 2 | Anteriormost medial point of the incisive foramen |
| L 3 | Posteriormost medial point of the incisive foramen |
| L 4 | Posteriormost medial point of the palate |
| L 5 | Anteriormost medial point of the foramen magnum |
| L 6 | Posteriormost medial point of the foramen magnum |
| L 7 | Lateralmost point of the foramen magnum (inner border of occipital condyle) |
|  | Lateralmost point of braincase, aligned with auditory bullae |
| L 9 | Anteriormost point of the auditory bullae |
| L 10 | Lateralmost point of the auditory bullae |
| L 11 | Posteriormost point of the auditory bullae |
| L 12 | Anteriormost point of external orbit |
| L 13 | Posteriormost point of internal orbit |
| L 14 | Anterior point of upper maxillary process |
| L 15 | Posteriormost point of the toothrow |
| L 16 | Anteriormost point of the toothrow |
| L 17 | Lingualmost point of the M1 |
| L 18 | Labialmost point of the M2 |
| L 19 | Lateralmost point of the incisive |

| Rodentia, Muridae, *Niviventer* | |
| --- | --- |
| L 1 | Anteriormost medial point of the nasal |
| L 2 | Anteriomost medial point of the premaxilla |
| L 3 | Anteriormost medial point of the incisive foramen |
| L 4 | Posteriormost medial point of the incisive foramen |
| L 5 | Posteriormost medial point of the palate |
| L 6 | Anteriormost medial point of the foramen magnum |
| L 7 | Lateralmost point of the foramen magnum (inner border of occipital condyle) |
| L 8 | Lateralmost point of braincase, aligned with auditory bullae |
| L 9 | Anteriormost point of the auditory bullae |
| L 10 | Posteriormost point of the auditory bullae |
| L 11 | Anteriormost point of external orbit |
| L 12 | Anteriormost point of internal orbit |
| L 13 | Anterior point of upper maxillary process |
| L 14 | Posteriormost point of the toothrow |
| L 15 | Anteriormost point of the toothrow |
| L 16 | Lingualmost point of the M1 |
| L 17 | Labialmost point of the M2 |
| L 18 | Lateralmost point of the maxilla |
| L 19 | Lateralmost point of the incisive |

**Table S3.** Results of sexual dimorphism analyses for the nine species used in this study. Sexual dimorphism in skull shape and size was tested via Hotelling’s T^2^ and t-test, respectively.

| Species | Shape dimorphism | Size dimorphism |
| --- | --- | --- |
| *Anourosorex squamipes* | T^2^ =1.16, *P* = 0.37 | t =0.16, df = 10, *P* =0.87 |
| *Soriculus nigrescens* | T^2^ = 1.307, *P* =1 | t = 0.892, df =10, *P* = 0.393 |
| *Uropsilus soricipes* | T^2^ = 1.307 , *P* =1 | t = 0.892, df = 10, *P* = 0.393 |
| *Eothenomys melanogaster* | T^2^= -1413, *P* = 0.84 | t = 0.071, df =10 , *P* = 0.05 |
| *Allactaga sibirica* | T^2^ = 0.83, *P* = 0.69 | t = 0.002, df = 12, *P* =0.99 |
| *Dipus sagitta* | T^2^ =2.18, *P* =0.25 | t = -0.09, df = 10, *P* =0.92 |
| *Apodemus chevrieri* | T^2^ = 0.563, *P* =1 | t = -0.744, df =10 , *P* = 0.473 |
| *Apodemus ilex* | T^2^ =1.527, *P* =0.415 | t = -0.555, df =10 , *P* =0.590 |
| *Niviventer confucianus* | T^2^ = 0.841, *P* =0.881 | t =-0.303, df = 10, *P* =0.767 |

**Table S4.** List of potential predators of small mammals in China, showing their altitudinal range. Asterisks indicate main predators of small terrestrial mammals

| **Group** | **Species** | **Elevation range** |
| --- | --- | --- |
| Birds | *Accipiter badius* (Gmelin, 1788) | 0-3000 |
| Birds | *Accipiter gentilis* (Linnaeus, 1758)* | 0-3400 |
| Birds | *Accipiter gularis* (Temminck & Schlegel, 1844) | 0-1800 |
| Birds | *Accipiter nisus* (Linnaeus, 1758)* | 0-4500 |
| Birds | *Accipiter soloensis* (Horsfield, 1821) | 0-1500 |
| Birds | *Accipiter trivirgatus* (Temminck, 1824)* | 0-2400 |
| Birds | *Accipiter virgatus* (Temminck, 1822) | 300-2200 |
| Birds | *Aegolius funereus* (Linnaeus, 1758)* | 0-2000 |
| Birds | *Aquila chrysaetos* (Linnaeus, 1758) | 0-4000 |
| Birds | *Aquila fasciata* (Vieillot, 1822) | 0-3000 |
| Birds | *Aquila heliaca* Savigny, 1809* | 0-1600 |
| Birds | *Aquila nipalensis* Hodgson, 1833* | 0-3000 |
| Birds | *Asio flammeus* (Pontoppidan, 1763)* | 0-4300 |
| Birds | *Asio otus* (Linnaeus, 1758)* | 0-2750 |
| Birds | *Athene noctua* (Scopoli, 1769)* | 0-2600 |
| Birds | *Aviceda jerdoni* (Blyth, 1842) | 200-1100 |
| Birds | *Bubo bubo* (Linnaeus, 1758)* | 0-4500 |
| Birds | *Bubo scandiacus* (Linnaeus, 1758)* | 0-300 |
| Birds | *Butastur indicus* (Gmelin, 1788)* | 0-2000 |
| Birds | *Butastur liventer* (Temminck, 1827)* | 0-1500 |
| Birds | *Butastur teesa* (Franklin, 1831)* | 0-1200 |
| Birds | *Buteo hemilasius* Temminck & Schlegel, 1844* | 0-5000 |
| Birds | *Buteo japonicus* Temminck & Schlegel, 1844* | 0-2500 |
| Birds | *Buteo lagopus* (Pontoppidan, 1763)* | 0-1200 |
| Birds | *Buteo refectus* Portenko, 1929* | 0-2500 |
| Birds | *Buteo rufinus* (Cretzschmar, 1827)* | 0-3900 |
| Birds | *Circaetus gallicus* (Gmelin, 1788) | 0-2300 |
| Birds | *Circus aeruginosus* (Linnaeus, 1758)* | 0-2000 |
| Birds | *Circus cyaneus* (Linnaeus, 1766)* | 0-3000 |
| Birds | *Circus macrourus* (Gmelin, 1770)* | 0-4000 |
| Birds | *Circus melanoleucos* (Pennant, 1769)* | 0-2100 |
| Birds | *Circus pygargus* (Linnaeus, 1758)* | 0-4000 |
| Birds | *Circus spilonotus* Kaup, 1847* | 0-2000 |
| Birds | *Clanga clanga* (Pallas, 1811)* | 0-1400 |
| Birds | *Elanus caeruleus* (Desfontaines, 1789)* | 0-3000 |
| Birds | *Falco cherrug* Gray, 1834* | 0-4700 |
| Birds | *Falco columbarius* Linnaeus, 1758 | 0-2600 |
| Birds | *Falco peregrinus* Tunstall, 1771 | 0-3300 |
| Birds | *Falco rusticolus* Linnaeus, 1758 | 0-1400 |
| Birds | *Falco tinnunculus* Linnaeus, 1758* | 0-3500 |
| Birds | *Glaucidium brodiei* (Burton, 1836) | 1350-2750 |
| Birds | *Glaucidium cuculoides* (Vigors, 1831) | 0-2700 |
| Birds | *Glaucidium passerinum* (Linnaeus, 1758)* | 250-2150 |
| Birds | *Haliaeetus albicilla* (Linnaeus, 1758) | 0-750 |
| Birds | *Haliaeetus leucogaster* (Gmelin, 1788) | 0-900 |
| Birds | *Haliastur indus* (Boddaert, 1783)* | 0-3000 |
| Birds | *Hieraaetus pennatus* (Gmelin, 1788)* | 0-3000 |
| Birds | *Ictinaetus malaiensis* (Temminck, 1822)* | 300-2700 |
| Birds | *Lophotriorchis kienerii* (Geoffroy Saint-Hilaire, 1835)* | 0-1500 |
| Birds | *Milvus migrans* (Boddaert, 1783)* | 0-4900 |
| Birds | *Nisaetus nipalensis* Hodgson, 1836 | 0-4000 |
| Birds | *Otus lettia* (Hodgson, 1836) | 0-2400 |
| Birds | *Otus semitorques* Temminck & Schlegel, 1844* | 0-900 |
| Birds | *Phodilus badius* (Horsfield, 1821)* | 0-2200 |
| Birds | *Strix aluco* Linnaeus, 1758* | 0-2350 |
| Birds | *Strix leptogrammica* Temminck, 1831* | 0-2500 |
| Birds | *Strix nebulosa* Forster, 1772* | 0-3200 |
| Birds | *Strix nivicolum* (Blyth, 1845)* | 0-2350 |
| Birds | *Strix uralensis* Pallas, 1771* | 450-1600 |
| Birds | *Surnia ulula* (Linnaeus, 1758)* | 0-2700 |
| Birds | *Tyto alba* (Scopoli, 1769)* | 0-4000 |
| Birds | *Tyto longimembris* (Jerdon, 1839)* | 0-2500 |
| Mammals | *Arctictis binturong* (Raffles, 1821) | 0-3000 |
| Mammals | *Arctogalidia trivirgata* (Gray, 1832) | 0-2100 |
| Mammals | *Arctonyx collaris* F.G. Cuvier, 1825 | 0-2300 |
| Mammals | *Canis lupus* Linnaeus, 1758* | 0-2400 |
| Mammals | *Catopuma temminckii* (Vigors & Horsfield, 1827)* | 0-3738 |
| Mammals | *Chrotogale owstoni* Thomas, 1912 | 100-2600 |
| Mammals | *Cuon alpinus* (Pallas, 1811) | 0-5300 |
| Mammals | *Felis bieti* Milne-Edwards, 1892* | 2500-5000 |
| Mammals | *Felis chaus* Schreber, 1777* | 0-4178 |
| Mammals | *Felis silvestris* Schreber, 1777* | 0-2250 |
| Mammals | *Gulo gulo* (Linnaeus, 1758) | 300-2400 |
| Mammals | *Herpestes javanicus* (É. Geoffroy Saint-Hilaire, 1818) | 0-1800 |
| Mammals | *Herpestes urva* (Hodgson, 1836) | 0-1800 |
| Mammals | *Lynx lynx* (Linnaeus, 1758) | 0-5500 |
| Mammals | *Martes flavigula* (Boddaert, 1785)* | 0-4510 |
| Mammals | *Martes foina* (Erxleben, 1777)* | 0-4200 |
| Mammals | *Martes zibellina* (Linnaeus, 1758)* | 20-2200 |
| Mammals | *Meles leucurus* (Hodgson, 1847)* | 0-3205 |
| Mammals | *Melogale moschata* (Gray, 1831)* | 0-1500 |
| Mammals | *Melogale personata* I. Geoffroy Saint-Hilaire, 1831* | 15-1520 |
| Mammals | *Mustela altaica* Pallas, 1811* | 80-4900 |
| Mammals | *Mustela erminea* Linnaeus, 1758* | 0-4050 |
| Mammals | *Mustela eversmanii* Lesson, 1827* | 0-5050 |
| Mammals | *Mustela kathiah* Hodgson, 1835* | 0-4000 |
| Mammals | *Mustela nivalis* Linnaeus, 1766* | 0-3860 |
| Mammals | *Mustela sibirica* Pallas, 1773* | 0-4875 |
| Mammals | *Mustela strigidorsa* Gray, 1853* | 90-2500 |
| Mammals | *Neofelis nebulosa* (Griffith, 1821) | 0-3000 |
| Mammals | *Nyctereutes procyonoides* (Gray, 1834)* | 0-3000 |
| Mammals | *Otocolobus manul* (Pallas, 1776)* | 450-5050 |
| Mammals | *Paguma larvata* (C.E.H. Smith, 1827) | 20-2700 |
| Mammals | *Panthera pardus* (Linnaeus, 1758) | 0-5200 |
| Mammals | *Panthera tigris* (Linnaeus, 1758) | 0-4500 |
| Mammals | *Panthera uncia* (Schreber, 1775) | 500-5800 |
| Mammals | *Paradoxurus hermaphroditus* (Pallas, 1777) | 0-2500 |
| Mammals | *Pardofelis marmorata* (Martin, 1837) | 0-2500 |
| Mammals | *Prionailurus bengalensis* (Kerr, 1792)* | 0-3240 |
| Mammals | *Prionodon pardicolor* Hodgson, 1841* | 150-3308 |
| Mammals | *Viverra megaspila* Blyth, 1862* | 0-800 |
| Mammals | *Viverra zibetha* Linnaeus, 1758* | 0-3080 |
| Mammals | *Viverricula indica* (É. Geoffroy Saint-Hilaire, 1803)* | 0-2500 |
| Mammals | *Vormela peregusna* (Güldenstädt, 1770)* | 0-3000 |
| Mammals | *Vulpes corsac* (Linnaeus, 1768)* | 0-2500 |
| Mammals | *Vulpes ferrilata* [Hodgson](https://species.wikimedia.org/wiki/Hodgson), 1842* | 2500-5200 |
| Mammals | *Vulpes vulpes* (Linnaues, 1758)* | 0-4500 |
| Snakes | *Azemiops feae* Boulenger, 1888* | 100-2200 |
| Snakes | *Bungarus fasciatus* (Schneider, 1801) | 20-2300 |
| Snakes | *Bungarus multicinctus* Blyth, 1861 | 0-1500 |
| Snakes | *Daboia siamensis* (Smith, 1917)* | 0-2000 |
| Snakes | *Deinagkistrodon acutus* (Günther, 1888)* | 0-1400 |
| Snakes | *Elaphe bimaculata* Schmidt, 1925* | 0-2240 |
| Snakes | *Elaphe carinata* (Günther, 1864)* | 100-2500 |
| Snakes | *Elaphe davidi* (Sauvage, 1884)* | 0-2000 |
| Snakes | *Elaphe schrenckii* Strauch, 1873* | 0-2000 |
| Snakes | *Elaphe zoigeensis* Huang et al, 2012* | 3200-3200 |
| Snakes | *Gloydius halys* (Pallas, 1776)* | 0-4000 |
| Snakes | *Naja atra* Cantor, 1842* | 70-1630 |
| Snakes | *Naja kaouthia* Lesson, 1831* | 0-1000 |
| Snakes | *Natrix natrix* (Linnaeus, 1758)* | 0-3600 |
| Snakes | *Natrix tessellata* (Laurenti, 1768)* | 0-2800 |
| Snakes | *Ophiophagus hannah* (Cantor, 1836) | 0-2000 |
| Snakes | *Ovophis monticola* (Günther, 1864)* | 600-2600 |
| Snakes | *Protobothrops jerdonii* (Günther, 1875)* | 1400-2300 |
| Snakes | *Protobothrops mangshanensis* (Zhao, 1990)* | 800-1300 |
| Snakes | *Protobothrops xiangchengensis* (Zhao, Jiang & Huang, 1978)* | 2750-3200 |
| Snakes | *Python bivittatus* Kuhl, 1820* | 10-4050 |
| Snakes | *Thermophis baileyi* (Wall, 1907) | 3000-4900 |
| Snakes | *Trimeresurus gramineus* (Shaw, 1802)* | 0-1400 |
| Snakes | *Vipera berus* (Linnaeus, 1758)* | 0-2700 |
| Snakes | *Vipera ursinii* (Bonaparte, 1835)* | 0-2700 |
| Snakes | *Viridovipera medoensis* (Zhao, 1977) | 1000-1400 |
| Snakes | *Viridovipera* *stejnegeri* (Schmidt, 1925) | 0-2000 |
| Snakes | *Xenopeltis* *hainanensis* Hu & Zhao, 1972* | 0-1200 |
| Snakes | *Xenopeltis* *unicolor* Reinwardt in Boie, 1827* | 0-1300 |

**Table S5.** Samples, localities, altitude (in metres) and GenBank accession numbers for cytochrome b sequences of *Niviventer confucianus* included in the present study. Protocols of DNA extraction and sequencing are detailed in Ge et al. (2018).

| **Field codes** | **Collection Locality** | **Altitude** | **GenBank Accession Number** |
| --- | --- | --- | --- |
| CA10129 | China, Sichuan, Luding, Gongga Mountain | 3200 | KP754592 |
| CA10303 | China, Sichuan, Luding, Gongga Mountain | 3200 | KP754593 |
| CA20267 | China, Sichuan, Luding, Gongga Mountain | 3200 | KF740032 |
| CA20270 | China, Sichuan, Luding, Gongga Mountain | 3200 | KF740033 |
| CA20308 | China, Sichuan, Luding, Gongga Mountain | 3200 | KF740034 |
| CB20193 | China, Sichuan, Luding, Gongga Mountain | 3200 | KP754595 |
| DA10015 | China, Sichuan, Luding, Gongga Mountain | 1600 | KF740036 |
| DA10029 | China, Sichuan, Luding, Gongga Mountain | 1600 | KF740042 |
| DA10030 | China, Sichuan, Luding, Gongga Mountain | 1600 | KF740043 |
| DA10034 | China, Sichuan, Luding, Gongga Mountain | 1600 | KF740044 |
| DA20035 | China, Sichuan, Luding, Gongga Mountain | 1600 | KF740045 |
| DA20036 | China, Sichuan, Luding, Gongga Mountain | 1600 | KF740046 |
| DA20037 | China, Sichuan, Luding, Gongga Mountain | 1600 | KF740047 |
| DA20039 | China, Sichuan, Luding, Gongga Mountain | 1600 | KF740049 |
| DA20040 | China, Sichuan, Luding, Gongga Mountain | 1600 | KF740050 |
| DA20041 | China, Sichuan, Luding, Gongga Mountain | 1600 | KP754598 |
| DA20044 | China, Sichuan, Luding, Gongga Mountain | 1600 | KF740052 |
| DA20052 | China, Sichuan, Luding, Gongga Mountain | 1600 | KF740054 |
| DA20054 | China, Sichuan, Luding, Gongga Mountain | 1600 | KP754599 |
| DB10021 | China, Sichuan, Luding, Gongga Mountain | 1600 | KF740056 |
| DB10022 | China, Sichuan, Luding, Gongga Mountain | 1600 | KF740057 |
| DB10025 | China, Sichuan, Luding, Gongga Mountain | 1600 | KF740058 |
| DB10029 | China, Sichuan, Luding, Gongga Mountain | 1600 | KF740042 |
| DB10032 | China, Sichuan, Luding, Gongga Mountain | 1600 | KF740060 |
| DB10033 | China, Sichuan, Luding, Gongga Mountain | 1600 | KF740061 |
| DB10036 | China, Sichuan, Luding, Gongga Mountain | 1600 | KF740062 |
| DB10037 | China, Sichuan, Luding, Gongga Mountain | 1600 | KP754601 |
| DB10038 | China, Sichuan, Luding, Gongga Mountain | 1600 | KP754602 |
| DB10039 | China, Sichuan, Luding, Gongga Mountain | 1600 | KP754603 |
| DB10040 | China, Sichuan, Luding, Gongga Mountain | 1600 | KP754604 |
| DB20024 | China, Sichuan, Luding, Gongga Mountain | 1600 | KP754605 |
| DB20025 | China, Sichuan, Luding, Gongga Mountain | 1600 | KP754606 |
| DB20026 | China, Sichuan, Luding, Gongga Mountain | 1600 | KF740063 |
| DB20027 | China, Sichuan, Luding, Gongga Mountain | 1600 | KP754607 |
| DB20028 | China, Sichuan, Luding, Gongga Mountain | 1600 | KF740064 |
| DB20029 | China, Sichuan, Luding, Gongga Mountain | 1600 | KF740065 |
| DB20030 | China, Sichuan, Luding, Gongga Mountain | 1600 | KF740066 |
| DB20031 | China, Sichuan, Luding, Gongga Mountain | 1600 | KF740067 |
| DB20032 | China, Sichuan, Luding, Gongga Mountain | 1600 | KF740068 |
| DB20033 | China, Sichuan, Luding, Gongga Mountain | 1600 | KF740069 |
| DB20047 | China, Sichuan, Luding, Gongga Mountain | 1600 | KP754609 |
| DB20048 | China, Sichuan, Luding, Gongga Mountain | 1600 | KF740070 |
| DB20049 | China, Sichuan, Luding, Gongga Mountain | 1600 | KP754610 |
| DB20050 | China, Sichuan, Luding, Gongga Mountain | 1600 | KF740071 |
| HA10215 | China, Sichuan, Luding, Gongga Mountain | 2800 | KF740089 |
| HA10217 | China, Sichuan, Luding, Gongga Mountain | 2800 | KF740090 |
| HA10220 | China, Sichuan, Luding, Gongga Mountain | 2800 | KP754611 |
| HA10221 | China, Sichuan, Luding, Gongga Mountain | 2800 | KP754612 |
| HA10225 | China, Sichuan, Luding, Gongga Mountain | 2800 | KF740091 |
| HA10227 | China, Sichuan, Luding, Gongga Mountain | 2800 | KP754613 |
| HA10228 | China, Sichuan, Luding, Gongga Mountain | 2800 | KP754614 |
| HA10231 | China, Sichuan, Luding, Gongga Mountain | 2800 | KF740092 |
| HA10235 | China, Sichuan, Luding, Gongga Mountain | 2800 | KF740094 |
| HA10239 | China, Sichuan, Luding, Gongga Mountain | 2800 | KF740095 |
| HA10242 | China, Sichuan, Luding, Gongga Mountain | 2800 | KF740096 |
| HA20320 | China, Sichuan, Luding, Gongga Mountain | 2800 | KF740097 |
| HA20325 | China, Sichuan, Luding, Gongga Mountain | 2800 | KF740098 |
| HA20327 | China, Sichuan, Luding, Gongga Mountain | 2800 | KF740099 |
| HA20336 | China, Sichuan, Luding, Gongga Mountain | 2800 | KP754616 |
| HA20339 | China, Sichuan, Luding, Gongga Mountain | 2800 | KP754617 |
| HA20343 | China, Sichuan, Luding, Gongga Mountain | 2800 | KP754618 |
| HB10260 | China, Sichuan, Luding, Gongga Mountain | 2800 | KF740100 |
| HB10261 | China, Sichuan, Luding, Gongga Mountain | 2800 | KF740101 |
| HB10262 | China, Sichuan, Luding, Gongga Mountain | 2800 | KF740102 |
| MA10001 | China, Sichuan, Luding, Gongga Mountain | 1200 | KP754619 |
| MA20003 | China, Sichuan, Luding, Gongga Mountain | 1200 | KF740178 |
| MA20004 | China, Sichuan, Luding, Gongga Mountain | 1200 | KP754623 |
| MA20005 | China, Sichuan, Luding, Gongga Mountain | 1200 | KF740179 |
| MA20008 | China, Sichuan, Luding, Gongga Mountain | 1200 | KP754624 |
| MA20009 | China, Sichuan, Luding, Gongga Mountain | 1200 | KP754625 |
| MA20010 | China, Sichuan, Luding, Gongga Mountain | 1200 | KP754626 |
| MA20011 | China, Sichuan, Luding, Gongga Mountain | 1200 | KP754627 |
| MA20012 | China, Sichuan, Luding, Gongga Mountain | 1200 | KF740181 |
| MA20017 | China, Sichuan, Luding, Gongga Mountain | 1200 | KF740182 |
| MA20018 | China, Sichuan, Luding, Gongga Mountain | 1200 | KF740183 |
| MB10008 | China, Sichuan, Luding, Gongga Mountain | 1200 | KF740185 |
| MB10014 | China, Sichuan, Luding, Gongga Mountain | 1200 | KP754628 |
| MB20001 | China, Sichuan, Luding, Gongga Mountain | 1200 | KF740186 |
| MB20002 | China, Sichuan, Luding, Gongga Mountain | 1200 | KF740187 |
| MB20006 | China, Sichuan, Luding, Gongga Mountain | 1200 | KP754629 |
| MB20007 | China, Sichuan, Luding, Gongga Mountain | 1200 | KF740188 |
| MB20013 | China, Sichuan, Luding, Gongga Mountain | 1200 | KF740189 |
| MB20014 | China, Sichuan, Luding, Gongga Mountain | 1200 | KF740190 |
| MB20015 | China, Sichuan, Luding, Gongga Mountain | 1200 | KP754631 |
| MB20020 | China, Sichuan, Luding, Gongga Mountain | 1200 | KP754632 |
| PA10119 | China, Sichuan, Luding, Gongga Mountain | 2400 | KF740195 |
| PA10123 | China, Sichuan, Luding, Gongga Mountain | 2400 | KF740196 |
| PA10126 | China, Sichuan, Luding, Gongga Mountain | 2400 | KF740197 |
| PA10127 | China, Sichuan, Luding, Gongga Mountain | 2400 | KF740198 |
| PA10167 | China, Sichuan, Luding, Gongga Mountain | 2400 | KP754633 |
| PA20135 | China, Sichuan, Luding, Gongga Mountain | 2400 | KF740199 |
| PA20140 | China, Sichuan, Luding, Gongga Mountain | 2400 | KP754634 |
| PA20158 | China, Sichuan, Luding, Gongga Mountain | 2400 | KP754635 |
| PA20164 | China, Sichuan, Luding, Gongga Mountain | 2400 | KF740200 |
| PB10093 | China, Sichuan, Luding, Gongga Mountain | 2400 | KP754636 |
| PB10094 | China, Sichuan, Luding, Gongga Mountain | 2400 | KF740201 |
| PB20139 | China, Sichuan, Luding, Gongga Mountain | 2400 | KF740202 |
| PB20140 | China, Sichuan, Luding, Gongga Mountain | 2400 | KF740203 |
| PB20152 | China, Sichuan, Luding, Gongga Mountain | 2400 | KF740204 |
| PB20173 | China, Sichuan, Luding, Gongga Mountain | 2400 | KP754639 |
| YA10052 | China, Sichuan, Luding, Gongga Mountain | 2000 | KP754640 |
| YA10053 | China, Sichuan, Luding, Gongga Mountain | 2000 | KP754641 |
| YA10055 | China, Sichuan, Luding, Gongga Mountain | 2000 | KP754642 |
| YA10057 | China, Sichuan, Luding, Gongga Mountain | 2000 | KP754643 |
| YA10146 | China, Sichuan, Luding, Gongga Mountain | 2000 | KF740227 |
| YA10147 | China, Sichuan, Luding, Gongga Mountain | 2000 | KP754644 |
| YA10148 | China, Sichuan, Luding, Gongga Mountain | 2000 | KF740228 |
| YA10150 | China, Sichuan, Luding, Gongga Mountain | 2000 | KP754645 |
| YA10156 | China, Sichuan, Luding, Gongga Mountain | 2000 | KF740229 |
| YA10160 | China, Sichuan, Luding, Gongga Mountain | 2000 | KF740230 |
| YA10168 | China, Sichuan, Luding, Gongga Mountain | 2000 | KP754646 |
| YA10169 | China, Sichuan, Luding, Gongga Mountain | 2000 | KP754647 |
| YA10172 | China, Sichuan, Luding, Gongga Mountain | 2000 | KP754649 |
| YA10175 | China, Sichuan, Luding, Gongga Mountain | 2000 | KP754650 |
| YA10176 | China, Sichuan, Luding, Gongga Mountain | 2000 | KP754651 |
| YA20056 | China, Sichuan, Luding, Gongga Mountain | 2000 | KF740232 |
| YA20070 | China, Sichuan, Luding, Gongga Mountain | 2000 | KF740233 |
| YA20071 | China, Sichuan, Luding, Gongga Mountain | 2000 | KF740234 |
| YA20072 | China, Sichuan, Luding, Gongga Mountain | 2000 | KF740235 |
| YA20092 | China, Sichuan, Luding, Gongga Mountain | 2000 | KF740236 |
| YA20094 | China, Sichuan, Luding, Gongga Mountain | 2000 | KF740237 |
| YA20099 | China, Sichuan, Luding, Gongga Mountain | 2000 | KF740238 |
| YA20100 | China, Sichuan, Luding, Gongga Mountain | 2000 | KF740239 |
| YA20105 | China, Sichuan, Luding, Gongga Mountain | 2000 | KF740240 |
| YA20108 | China, Sichuan, Luding, Gongga Mountain | 2000 | KP754652 |
| YA20119 | China, Sichuan, Luding, Gongga Mountain | 2000 | KF740241 |
| YA20121 | China, Sichuan, Luding, Gongga Mountain | 2000 | KF740242 |
| YB10041 | China, Sichuan, Luding, Gongga Mountain | 2000 | KP754653 |
| YB10042 | China, Sichuan, Luding, Gongga Mountain | 2000 | KF740243 |
| YB10043 | China, Sichuan, Luding, Gongga Mountain | 2000 | KF740243 |
| YB10050 | China, Sichuan, Luding, Gongga Mountain | 2000 | KF740244 |
| YB10058 | China, Sichuan, Luding, Gongga Mountain | 2000 | KF740245 |
| YB10059 | China, Sichuan, Luding, Gongga Mountain | 2000 | KF740246 |
| YB10063 | China, Sichuan, Luding, Gongga Mountain | 2000 | KP754655 |
| YB10070 | China, Sichuan, Luding, Gongga Mountain | 2000 | KF740247 |
| YB10071 | China, Sichuan, Luding, Gongga Mountain | 2000 | KP754656 |
| YB10072 | China, Sichuan, Luding, Gongga Mountain | 2000 | KF740248 |
| YB10075 | China, Sichuan, Luding, Gongga Mountain | 2000 | KF740249 |
| YB10083 | China, Sichuan, Luding, Gongga Mountain | 2000 | KF740250 |
| YB10085 | China, Sichuan, Luding, Gongga Mountain | 2000 | KF740251 |
| YB10173 | China, Sichuan, Luding, Gongga Mountain | 2000 | KP754657 |
| YB20058 | China, Sichuan, Luding, Gongga Mountain | 2000 | KF740252 |
| YB20063 | China, Sichuan, Luding, Gongga Mountain | 2000 | KF740253 |
| YB20073 | China, Sichuan, Luding, Gongga Mountain | 2000 | KF740254 |
| YB20074 | China, Sichuan, Luding, Gongga Mountain | 2000 | KF740255 |
| YB20076 | China, Sichuan, Luding, Gongga Mountain | 2000 | KF740256 |
| YB20079 | China, Sichuan, Luding, Gongga Mountain | 2000 | KP754659 |
| YB20080 | China, Sichuan, Luding, Gongga Mountain | 2000 | KP754660 |
| YB20083 | China, Sichuan, Luding, Gongga Mountain | 2000 | KP754661 |
| YB20085 | China, Sichuan, Luding, Gongga Mountain | 2000 | KF740258 |
| YB20087 | China, Sichuan, Luding, Gongga Mountain | 2000 | KP754662 |
| YB20088 | China, Sichuan, Luding, Gongga Mountain | 2000 | KF740259 |
| YB20089 | China, Sichuan, Luding, Gongga Mountain | 2000 | KP754663 |
| YB20090 | China, Sichuan, Luding, Gongga Mountain | 2000 | KF740260 |
| YB20091 | China, Sichuan, Luding, Gongga Mountain | 2000 | KF740261 |
| YB20110 | China, Sichuan, Luding, Gongga Mountain | 2000 | KF740262 |
| YB20111 | China, Sichuan, Luding, Gongga Mountain | 2000 | KP754665 |
| AZ016 | China, Sichuan, Puge, Luoji Mountain | 3400 | KY304359 |
| AZ017 | China, Sichuan, Puge, Luoji Mountain | 3400 | KF739997 |
| AZ018 | China, Sichuan, Puge, Luoji Mountain | 3400 | KF739998 |
| AZ036 | China, Sichuan, Puge, Luoji Mountain | 3400 | KF739999 |
| AZ037 | China, Sichuan, Puge, Luoji Mountain | 3400 | KF740000 |
| AZ038 | China, Sichuan, Puge, Luoji Mountain | 3400 | KF740001 |
| AZ057 | China, Sichuan, Puge, Luoji Mountain | 3400 | KF740002 |
| AZ069 | China, Sichuan, Puge, Luoji Mountain | 3400 | KF740003 |
| AZ071 | China, Sichuan, Puge, Luoji Mountain | 3400 | KF740004 |
| AZ073 | China, Sichuan, Puge, Luoji Mountain | 3400 | KF740006 |
| BBD001 | China, Sichuan, Puge, Luoji Mountain | 2600 | KF740008 |
| BBD002 | China, Sichuan, Puge, Luoji Mountain | 2600 | KF740009 |
| BBD009 | China, Sichuan, Puge, Luoji Mountain | 2600 | KY304360 |
| BBD010 | China, Sichuan, Puge, Luoji Mountain | 2600 | KY304361 |
| BBD014 | China, Sichuan, Puge, Luoji Mountain | 2600 | KY304362 |
| BBD020 | China, Sichuan, Puge, Luoji Mountain | 2600 | KY304363 |
| BBD027 | China, Sichuan, Puge, Luoji Mountain | 2600 | KF740013 |
| BBD046 | China, Sichuan, Puge, Luoji Mountain | 2600 | KF740014 |
| BBD047 | China, Sichuan, Puge, Luoji Mountain | 2600 | KF740015 |
| BBD048 | China, Sichuan, Puge, Luoji Mountain | 2600 | KF740016 |
| BBD049 | China, Sichuan, Puge, Luoji Mountain | 2600 | KF740017 |
| BBD050 | China, Sichuan, Puge, Luoji Mountain | 2600 | KF740018 |
| BBD052 | China, Sichuan, Puge, Luoji Mountain | 2600 | KF740019 |
| BBD054 | China, Sichuan, Puge, Luoji Mountain | 2600 | KF740020 |
| BBD055 | China, Sichuan, Puge, Luoji Mountain | 2600 | KY304364 |
| BBD056 | China, Sichuan, Puge, Luoji Mountain | 2600 | KF740021 |
| BBD057 | China, Sichuan, Puge, Luoji Mountain | 2600 | KF740022 |
| BBD058 | China, Sichuan, Puge, Luoji Mountain | 2600 | KF740023 |
| BBD059 | China, Sichuan, Puge, Luoji Mountain | 2600 | KF740024 |
| BBD061 | China, Sichuan, Puge, Luoji Mountain | 2600 | KF740025 |
| BBD072 | China, Sichuan, Puge, Luoji Mountain | 2600 | KF740026 |
| BBD073 | China, Sichuan, Puge, Luoji Mountain | 2600 | KY304365 |
| BBD074 | China, Sichuan, Puge, Luoji Mountain | 2600 | KF740027 |
| JCB007 | China, Sichuan, Puge, Luoji Mountain | 4150 | KY304366 |
| LZA004 | China, Sichuan, Puge, Luoji Mountain | 3000 | KF740156 |
| LZA007 | China, Sichuan, Puge, Luoji Mountain | 3000 | KF740157 |
| LZA008 | China, Sichuan, Puge, Luoji Mountain | 3000 | KF740158 |
| LZA009 | China, Sichuan, Puge, Luoji Mountain | 3000 | KF740159 |
| LZA010 | China, Sichuan, Puge, Luoji Mountain | 3000 | KY304367 |
| LZA013 | China, Sichuan, Puge, Luoji Mountain | 3000 | KY304368 |
| LZA024 | China, Sichuan, Puge, Luoji Mountain | 3000 | KY304369 |
| LZA025 | China, Sichuan, Puge, Luoji Mountain | 3000 | KY304370 |
| LZA027 | China, Sichuan, Puge, Luoji Mountain | 3000 | KF740160 |
| LZA029 | China, Sichuan, Puge, Luoji Mountain | 3000 | KF740161 |
| LZA030 | China, Sichuan, Puge, Luoji Mountain | 3000 | KF740162 |
| LZA031 | China, Sichuan, Puge, Luoji Mountain | 3000 | KF740163 |
| LZA032 | China, Sichuan, Puge, Luoji Mountain | 3000 | KY304371 |
| LZA033 | China, Sichuan, Puge, Luoji Mountain | 3000 | KY304372 |
| LZA034 | China, Sichuan, Puge, Luoji Mountain | 3000 | KY304373 |
| LZA035 | China, Sichuan, Puge, Luoji Mountain | 3000 | KY304374 |
| LZA036 | China, Sichuan, Puge, Luoji Mountain | 3000 | KY304375 |
| LZA042 | China, Sichuan, Puge, Luoji Mountain | 3000 | KF740168 |
| LZA043 | China, Sichuan, Puge, Luoji Mountain | 3000 | KY304376 |
| LZA045 | China, Sichuan, Puge, Luoji Mountain | 3000 | KY304377 |
| LZA046 | China, Sichuan, Puge, Luoji Mountain | 3000 | KY304378 |
| LZA048 | China, Sichuan, Puge, Luoji Mountain | 3000 | KY304379 |
| LZA049 | China, Sichuan, Puge, Luoji Mountain | 3000 | KY304380 |
| LZA068 | China, Sichuan, Puge, Luoji Mountain | 3000 | KY304381 |
| LZA069 | China, Sichuan, Puge, Luoji Mountain | 3000 | KY304382 |
| LZA071 | China, Sichuan, Puge, Luoji Mountain | 3000 | KY304383 |
| LZA072 | China, Sichuan, Puge, Luoji Mountain | 3000 | KY304384 |
| LZA073 | China, Sichuan, Puge, Luoji Mountain | 3000 | KY304385 |
| LZA074 | China, Sichuan, Puge, Luoji Mountain | 3000 | KY304386 |
| SWS002 | China, Sichuan, Puge, Luoji Mountain | 2200 | KF740213 |
| SWS037 | China, Sichuan, Puge, Luoji Mountain | 2200 | KF740214 |
| SWS048 | China, Sichuan, Puge, Luoji Mountain | 2200 | KY304387 |
| SWS050 | China, Sichuan, Puge, Luoji Mountain | 2200 | KY304388 |
| TMG006 | China, Sichuan, Puge, Luoji Mountain | 1800 | KY304389 |
| TMG009 | China, Sichuan, Puge, Luoji Mountain | 1800 | KY304390 |
| TMG029 | China, Sichuan, Puge, Luoji Mountain | 1800 | KY304391 |
| WL14017 | China, Sichuan, Wolong Mountain | 1930 | KY304444 |
| WL14019 | China, Sichuan, Wolong Mountain | 1930 | KY304445 |
| WL14069 | China, Sichuan, Wolong Mountain | 2800 | KY304446 |
| WL14076 | China, Sichuan, Wolong Mountain | 2800 | KY304447 |
| WL14078 | China, Sichuan, Wolong Mountain | 2800 | KY304448 |
| WL14080 | China, Sichuan, Wolong Mountain | 2800 | KY304449 |
| WL14113 | China, Sichuan, Wolong Mountain | 3003 | KY304450 |
| WL14115 | China, Sichuan, Wolong Mountain | 3003 | KY304451 |
| WL14120 | China, Sichuan, Wolong Mountain | 2800 | KY304452 |
| WL14125 | China, Sichuan, Wolong Mountain | 2800 | KY304453 |
| WL14129 | China, Sichuan, Wolong Mountain | 3003 | KY304454 |
| WL14151 | China, Sichuan, Wolong Mountain | 2500 | KY304455 |
| WL14195 | China, Sichuan, Wolong Mountain | 2500 | KY304456 |
| WL14240 | China, Sichuan, Wolong Mountain | 1800 | KY304457 |
| WL14243 | China, Sichuan, Wolong Mountain | 1800 | KY304458 |
| WL14290 | China, Sichuan, Wolong Mountain | 2200 | KY304459 |
| WL14294 | China, Sichuan, Wolong Mountain | 2200 | KY304460 |
| WL14316 | China, Sichuan, Wolong Mountain | 2200 | KY304461 |
| WL14318 | China, Sichuan, Wolong Mountain | 1550 | KY304462 |
| WL14321 | China, Sichuan, Wolong Mountain | 1550 | KY304463 |
| WL14325 | China, Sichuan, Wolong Mountain | 1550 | KY304464 |
| WL14339 | China, Sichuan, Wolong Mountain | 1550 | KY304466 |

**Table S6.** *P* values of Tukey test (for size) and *P* values of Pairwise distances between group means (for shape) across elevation zones (see main text for zonation definition). Numbers in boldface represent significant values (p<0.05 based on a permutation test with 10000 randomizations).

| Species | Size | | | Shape | | |
| --- | --- | --- | --- | --- | --- | --- |
|  | Low *vs* Middle | Low *vs* High | Middle *vs* High | Low *vs* Middle | Low *vs* High | Middle *vs* High |
| *A. squamipes* | **0.000** | - | - | **0.008** | - | - |
| *S. nigrescens* | 0.76 | 0.87 | 0.99 | **0.05** | **0.013** | 1 |
| *U. soricipes* | 0.83 | 0.58 | 0.88 | 0.066 | 0.992 | **0.01** |
| *E. melanogaster* | **0.000** | - | - | **0.016** | - | - |
| *A. sibirica* | **0.000** | **0.000** | 0.93 | **0.000** | **0.000** | **0.00** |
| *D. sagitta* | 0.87 | 0.53 | 0.54 | **0.000** | **0.000** | **0.00** |
| *A. chevrieri* | 0.27 | 0.36 | **0.05** | **0.05** | 1 | 0.34 |
| *A. ilex* | **0.03** | 0.18 | 0.96 | 0.21 | 1 | 1 |
| *N. confucianus* | 0.80 | **0.09** | 0.29 | 0.12 | **0.09** | 0.43 |

**Table S7.** Full statistics of model average considering models ∆AICc ≤ 6 for each species, including standard error (SE), relative importance of each parameter (Importance), number of models in which the factor occurs (N models) and 95% confidence interval (95% CI). Shape^1^ represents the score of regression of Procrustes coordinates on the log of centroid size. Shape^2^ represents the first principal component of Procrustes coordinates. (^2) indicates that the quadratic term of the parameter was used in the analysis. NPP, net primary productivity.

| **Parameter** | **Estimate** | **SE** | **Importance** | **N models** | **95% CI** | |
| --- | --- | --- | --- | --- | --- | --- |
| ***Anourosorex squamipes*** | |  |  |  |  |  |
| **Size** |  |  |  |  |  |  |
| Intercept | 3426 | 18.02 | - | - | 3386.92 | 3458.61 |
| Altitude | -3.64 | 13.72 | 0.08 | 139 | -87.24 | -4.03 |
| Intraspecific Competition | -0.01 | 0.52 | <0.01 | 3 | -38.11 | 15.67 |
| Interspecific Competition | -0.05 | 1.32 | <0.01 | 10 | -51.06 | 16.1 |
| Predator Richness | 33.63 | 44.36 | 0.39 | 563 | 37.55 | 133.1 |
| Main Predator Richness | 27.86 | 37.66 | 0.38 | 767 | 30.14 | 114.81 |
| NPP | -6.67 | 19.28 | 0.13 | 245 | -97.54 | -7.49 |
| Annual Temperature | 6.63 | 19.8 | 0.12 | 229 | 9.32 | 102.33 |
| Annual Precipitation | 0.34 | 5.71 | 0.01 | 28 | -56.88 | 112.35 |
| Precipitation Seasonality | -0.21 | 3 | 0.01 | 14 | -65.74 | -2.55 |
| **Shape^1^** |  |  |  |  |  |  |
| Intercept | -0.0002 | 0.01 | - | - | -0.002 | 0.002 |
| NPP | -0.0004 | 0.001 | 0.16 | 2 | -0.004 | -0.001 |
| Main Predator Richness | 0.0003 | 0.0009 | 0.10 | 2 | 0.001 | 0.004 |
| ***Soriculus nigrescens*** | |  |  |  |  |  |
| **Size** |  |  |  |  |  |  |
| Intercept | 2952.46 | 24.77 | - | - | 2914.25 | 2957.11 |
| Altitude | 1.16 | 6.02 | 0.05 | 2 | -30.07 | 97.41 |
| NPP | -3.16 | 12.83 | 0.07 | 1 | -18.26 | 47.86 |
| Annual Temperature | -0.39 | 3.57 | 0.02 | 1 | -47.8 | 108.8 |
| Annual Precipitation | -0.57 | 4.53 | 0.02 | 1 | -126.2 | 45.4 |
| Precipitation Seasonality | -0.54 | 4.26 | 0.02 | 1 | -33.0 | 10.83 |
| ***Uropsilus soricipes*** | |  |  |  |  |  |
| **Size** |  |  |  |  |  |  |
| Intercept | 2516.61 | 7.12 | - | - | 2501.73 | 2531.5 |
| Altitude | -111.64 | 172 | 0.52 | 23 | -588.75 | 157.91 |
| Intraspecific Competition | 0.22 | 5.49 | 0.09 | 8 | -104.19 | 143.34 |
| Interspecific Competition | 6.36 | 11.34 | 0.30 | 18 | 0.32 | 42.71 |
| Predator Richness | 19.88 | 34.6 | 0.31 | 13 | 0.21 | 128.57 |
| Main Predator Richness | 20.68 | 100.9 | 0.33 | 19 | -271.81 | 397 |
| NPP | 0.33 | 4.24 | 0.05 | 4 | -30.06 | 44.68 |
| Annual Temperature | -147.2 | 237.03 | 0.57 | 26 | -787.09 | 270.97 |
| Temperature Seasonality | -31.59 | 31.28 | 0.64 | 28 | -100.57 | 2.56 |
| Annual Precipitation | -14.48 | 17.82 | 0.45 | 27 | -56.49 | -7.53 |
| Precipitation Seasonality | 12.89 | 19.96 | 0.36 | 20 | 0.82 | 69.89 |
| **Shape^1^** |  |  |  |  |  |  |
| Intercept | 0.000 | 0.001 | - | - | -0.003 | 0.003 |
| Interspec. comp. | 0.004 | 0.001 | 0.1 | 1 | 0.001 | 0.007 |
| ***Eothenomys melanogaster*** | |  |  |  |  |  |
| **Size** |  |  |  |  |  |  |
| Intercept | 2863.15 | 17.43 | - | - | 2826.85 | 2899.45 |
| Intraspecific Competition | 175.73 | 34.59 | 1 | 4 | 104.24 | 247.22 |
| Altitude | -1.79 | 10 | 0.04 | 1 | -95.64 | 14.41 |
| **Shape^1^** |  |  |  |  |  |  |
| Intercept | -0.002 | 0.002 | - | - | -0.008 | 0.002 |
| Annual Temperature | 0.00005 | 0.0006 | 0.01 | 1 | 0.0003 | 0.01 |
| Altitude | -0.00004 | 0.0005 | 0.01 | 1 | -0.01 | -0.0001 |
| ***Allactaga sibirica*** | |  |  |  |  |  |
| **Size** |  |  |  |  |  |  |
| Intercept | 2838.43 | 11.54 | - | - | 2815.51 | 2861.36 |
| Altitude | 14.53 | 34.51 | 0.21 | 43 | -18.02 | 155.06 |
| Intraspecific Competition | 2.64 | 8.52 | 0.11 | 24 | 1.34 | 47.81 |
| Interspecific Competition | 0.68 | 4.05 | 0.04 | 15 | -4.62 | 39.68 |
| Predator Richness | -0.51 | 5.1 | 0.03 | 10 | -69.1 | 29.53 |
| NPP | -2.03 | 8.79 | 0.07 | 15 | -64.39 | 5.8 |
| Annual Temperature | 0.2 | 2.68 | 0.01 | 3 | -4.19 | 56.73 |
| Temperature Seasonality | 70.42 | 39.41 | 0.67 | 132 | 7.44 | 145.41 |
| Annual Precipitation | 2.07 | 15.28 | 0.06 | 24 | -68.19 | 142.63 |
| Precipitation Seasonality^2 | 85.26 | 100.22 | 0.69 | 62 | -65.28 | 320.86 |
| Precipitation Seasonality | 11.33 | 102.85 | 0.45 | 100 | 268.42 | 316.55 |
| **Shape^2^** |  |  |  |  |  |  |
| Intercept | 0.000 | 0.001 | 0.15 | - | -0.002 | 0.002 |
| Altitude | -0.009 | 0.002 | 0.92 | 2 | -0.012 | -0.008 |
| Annual Precipitation | 0.000 | 0.002 | 0.08 | 1 | 0.008 | 0.014 |
| Precipitation Seasonality^2 | 0.004 | 0.003 | 0.61 | 1 | 0.005 | 0.009 |
| Precipitation Seasonality | 0.002 | 0.003 | 0.31 | 1 | 0.005 | 0.008 |
| ***Dipus sagitta*** |  |  |  |  |  |  |
| **Size** |  |  |  |  |  |  |
| Intercept | 2589.37 | 12.14 | - | - | 2565.19 | 2613.55 |
| Altitude(^2) | 0.77 | 4.07 | 0.05 | 1 | -5.0 | 35.79 |
| Intraspecific Competition | 3.17 | 12.48 | 0.06 | 1 | 31.56 | 69.53 |
| NPP | 3.02 | 11.8 | 0.06 | 1 | 34.55 | 61.75 |
| Annual Precipitation | 70.67 | 20.75 | 0.94 | 2 | 55.22 | 95.58 |
| **Shape^2^** |  |  |  |  |  |  |
| Intercept | 0.002 | 0.004 | - | - | -0.005 | 0.01 |
| Altitude(^2) | 0.0002 | 0.001 | 0.04 | 1 | 0.002 | 0.01 |
| ***Apodemus chevrieri*** | |  |  |  |  |  |
| **Size** |  |  |  |  |  |  |
| Intercept | 3474.83 | 22.3 | - | - | 3429.08 | 3520.58 |
| Altitude | 4.84 | 23.02 | 0.11 | 7 | -68.14 | 152.52 |
| Intraspecific Competition | -0.48 | 7.69 | 0.03 | 2 | -105.27 | 69.27 |
| Interspecific Competition | 0.14 | 3.11 | 0.01 | 1 | -38.7 | 60.04 |
| Predator Richness | 114 | 53.04 | 0.94 | 37 | 27.71 | 213.96 |
| Main Predator Richness | 1.3 | 16.89 | 0.07 | 5 | -102.09 | 137.38 |
| NPP | -2.46 | 13.6 | 0.06 | 4 | -120.97 | 34.01 |
| Annual Temperature | -10.53 | 31.85 | 0.18 | 10 | -166.51 | 45.55 |
| Temperature Seasonality | 0.94 | 7.78 | 0.03 | 3 | -36.65 | 92.94 |
| Annual Precipitation | -10.61 | 25.27 | 0.19 | 6 | -114.94 | 3.95 |
| Precipitation Seasonality | 5.26 | 18.94 | 0.1 | 7 | -17.51 | 119.2 |
| **Shape^1^** |  |  |  |  |  |  |
| Intercept | -0.004 | 0.003 | - | - | -0.01 | 0.002 |
| Predator Richness | 0.001 | 0.003 | 0.18 | 1 | 0.004 | 0.015 |
| Main Predator Richness | 0.0006 | 0.002 | 0.08 | 1 | 0.002 | 0.01 |
| **Shape^2^** |  |  |  |  |  |  |
| Intercept | 0.004 | 0.001 | - | - | 0.001 | 0.008 |
| Predator Richness | -0.001 | 0.002 | 0.33 | 1 | -0.008 | -0.003 |
| Main Predator Richness | -0.0007 | 0.001 | 0.14 | 1 | -0.007 | -0.00 |
| ***Apodemus ilex*** |  |  |  |  |  |  |
| **Size** |  |  |  |  |  |  |
| Intercept | 3339.69 | 10.02 | - | - | 3319.69 | 3359.69 |
| Altitude | -14.34 | 38.2 | 0.21 | 77 | -181.76 | 42.29 |
| Intraspecific Competition | -0.1 | 1.69 | 0.01 | 8 | -32.54 | 17.46 |
| Interspecific Competition | 0.2 | 3.58 | 0.03 | 15 | -32.81 | 48.46 |
| Predator Richness | 35.39 | 52.66 | 0.52 | 124 | -41.96 | 178.07 |
| Main Predator Richness | -2.9 | 45.89 | 0.38 | 94 | -153.16 | 138.01 |
| NPP | -15.18 | 33.48 | 0.37 | 109 | -128.6 | 47.09 |
| Annual Temperature | -9.41 | 30.35 | 0.17 | 64 | -161.19 | 50.51 |
| Temperature Seasonality | 16.31 | 27.19 | 0.36 | 84 | -7.59 | 99.17 |
| Annual Precipitation | 8.58 | 18.16 | 0.25 | 81 | -7.01 | 75.73 |
| Precipitation Seasonality | 26.94 | 35.09 | 0.51 | 138 | -10.47 | 117.13 |
| **Shape^2^** |  |  |  |  |  |  |
| Intercept | 0.001 | 0.002 | - | - | -0.002 | 0.005 |
| NPP | 0.001 | 0.002 | 0.24 | 1 | 0.003 | 0.008 |
| Temperature Seasonality | -0.0004 | 0.001 | 0.09 | 1 | -0.007 | -0.002 |
| Interspecific Competition | 0.0002 | 0.001 | 0.05 | 1 | 0.002 | 0.008 |
| ***Niviventer confucianus*** | |  |  |  |  |  |
| **Size** |  |  |  |  |  |  |
| Intercept | 4412.25 | 27.58 | - | - | 4356.77 | 4467.71 |
| Altitude | -3.59 | 19.84 | 0.11 | 21 | -136.17 | 69.9 |
| Intraspecific Competition | 1.63 | 19.65 | 0.1 | 16 | -102.45 | 134.1 |
| Interspecific Competition | 2.38 | 13.21 | 0.08 | 16 | -44.82 | 102.22 |
| Predator Richness | 5.05 | 21.51 | 0.12 | 24 | -54.72 | 136.28 |
| Main Predator Richness | 3.25 | 17.01 | 0.1 | 17 | -55.47 | 120.74 |
| NPP | -2.34 | 10.82 | 0.08 | 14 | -81.26 | 23.39 |
| Annual Temperature | -1.38 | 20.66 | 0.11 | 23 | -137.28 | 111.13 |
| Temperature Seasonality | 3.1 | 14.76 | 0.09 | 15 | -39.74 | 106.55 |
| Annual Precipitation | 0.52 | 6.24 | 0.04 | 6 | -44.2 | 70.84 |
| Precipitation Seasonality | 62.57 | 36.09 | 0.85 | 110 | 19.21 | 127.74 |

**Table S8.** Analysis of molecular variance of *Niviventer confucianus* comparing elevation zones (low, midde and high) in three mountains of China. *P* values are based on 10000 permutations. Boldface represent significance at 5%.

| Gongga Mountain | | | | |
| --- | --- | --- | --- | --- |
|  | Variation | % | Ф-value | *P* |
| Among elevation zones | 0.105 | 1.56 | 0.015 | 0.21 |
| Among pop/Elev.zone | 0.154 | 2.28 | 0.023 | 0.072 |
| Within populations | 6.515 | 96.16 | 0.038 | **0.026** |
| Luoji Mountain | | | | |
|  | Variation | % | Ф-value | *P* |
| Among elevation zones | 0.55 | 10.08 | 0.10 | 0.12 |
| Among pop/Elev.zone | 0.218 | 3.97 | 0.044 | 0.064 |
| Within populations | 4.728 | 85.95 | 0.14 | **0.018** |
| Wolong Mountain | | | | |
|  | Variation | % | Ф-value | *P* |
| Among elevation zones | 1.847 | 24.28 | 0.24 | 0.19 |
| Among pop/Elev.zone | 1.20 | 15.81 | 0.20 | 0.04 |
| Within populations | 4.55 | 59.91 | 0.40 | **0.0009** |

**Table S9.** Genetic diversity of *Niviventer* *confucianus* populations based on mtDNA distributed in three mountains of China. Elevation zone (Elev. zone), populations (Pop.), numbers of individuals (n), number of polymorphic (segregating) sites (s), number of haplotypes (nh), haplotype diversity (h), mean number of pairwise nucleotide differences (k), nucleotide diversity (Pi).

| Mountain | Elev. zone | Pop. | n | s | nh | h | K | Pi (%) |
| --- | --- | --- | --- | --- | --- | --- | --- | --- |
| Gongga |  | Total | 160 | 59 | 42 | 0.91 | 10.2 | 0.9 |
|  | Low | 1200m | 22 | 41 | 15 | 0.95 | 11.2 | 1 |
|  |  | 1600m | 39 | 33 | 16 | 0.85 | 10.2 | 0.9 |
|  |  | 2000m | 57 | 35 | 19 | 0.88 | 8.9 | 0.8 |
|  | Middle | 2400m | 15 | 35 | 10 | 0.92 | 11.6 | 1.1 |
|  |  | 2800m | 20 | 36 | 11 | 0.91 | 10.94 | 1 |
|  | High | 3200m | 7 | 13 | 5 | 0.85 | 3.9 | 0.3 |
| Luoji |  | Total | 69 | 53 | 24 | 0.88 | 4.21 | 0.3 |
|  | Low | 1800m | 3 | 4 | 2 | 0.6 | 2.6 | 0.2 |
|  | Middle | 2200m | 4 | 9 | 3 | 0.83 | 4.6 | 0.4 |
|  |  | 2600m | 23 | 19 | 10 | 0.73 | 2.4 | 0.2 |
|  | High | 3000m | 29 | 26 | 8 | 0.74 | 4.78 | 0.4 |
|  |  | 3400m | 10 | 18 | 7 | 0.86 | 4.2 | 0.3 |
| Wolong |  | Total | 23 | 40 | 11 | 0.91 | 12.49 | 1.1 |
|  | Low | 1500m | 5 | 0 | 1 | 0 | 0 | 0 |
|  |  | 1900m | 4 | 13 | 2 | 0.66 | 8.66 | 0.7 |
|  | Middle | 2200m | 3 | 11 | 3 | 1 | 7.33 | 0.6 |
|  |  | 2500m | 2 | 15 | 2 | 1 | 15 | 1.3 |
|  | High | 2800m | 6 | 23 | 3 | 0.73 | 11.93 | 1 |
|  |  | 3000m | 3 | 20 | 2 | 0.66 | 13.3 | 1.1 |

**Table S10**. Pairwise estimates of F_ST_ between pair of populations of *Niviventer confucianus* in Gongga, Luoji and Wolong Mountains, China. *P* values based on 10000 permutations. Boldface represent significance at 5%.

| **Gongga Mountain** | | | | | | |
| --- | --- | --- | --- | --- | --- | --- |
| **Elevation zone** | **Population** | **1200m** | **1600m** | **2000m** | **2400m** | **2800m** |
| **Low** | 1200 m | - |  |  |  |  |
|  | 1600 m | 0.03 | - |  |  |  |
|  | 2000 m | **0.04** | 0.02 | - |  |  |
| **Middle** | 2400 m | 0.02 | 0.00 | 0.00 | - |  |
|  | 2800 m | 0.00 | 0.01 | 0.00 | 0.03 | - |
| **High** | 3200 m | **0.26** | **0.13** | **0.13** | **0.10** | **0.15** |
| **Luoji Mountain** | | | | | | |
| **Elevation zone** | **Population** | **1500m** | **2200m** | **2600m** | **3000m** | **3400m** |
| **Low** | 1800 m | - |  |  |  |  |
| **Middle** | 2200 m | 0.11 | - |  |  |  |
|  | 2600 m | 0.08 | 0.01 | - |  |  |
| **High** | 3000 m | **0.43** | 0.02 | **0.14** | - |  |
|  | 3400 m | 0.28 | 0.00 | 0.02 | **0.09** | - |
| **Wolong Mountain** | | | | | | |
| **Elevation zone** | **Population** | **1500m** | **1900m** | **2200m** | **2500m** | **2800m** |
| **Low** | 1500 m | - |  |  |  |  |
|  | 1900 m | **0.606** | - |  |  |  |
| **Middle** | 2200 m | **0.87** | 0.27 | - |  |  |
|  | 2500 m | 0.77 | 0.12 | 0.03 | - |  |
| **High** | 2800 m | **0.59** | 0.092 | 0.03 | 0.00 | - |
|  | 3000 m | **0.77** | 0.20 | 0.15 | 0.2 | 0.03 |
